# Fibro-adipogenic progenitors enhance functional and structural properties of human 3D tissue engineered skeletal muscles

**DOI:** 10.1177/20417314261441552

**Published:** 2026-04-25

**Authors:** Roy Augustinus, Lotte A. de Ridder, Dongxu Zheng, Marnix Franken, Judit Balog, Patrick J. van der Vliet, Alessandro Iuliano, Remko Goossens, Johanna I. Hamel, W. W. M. Pim Pijnappel, Jessica C. de Greef, Silvère M. van der Maarel

**Affiliations:** 1 Department of Human Genetics, Leiden University Medical Center, The Netherlands; 2 Department of Clinical Genetics, Erasmus University Medical Center, Rotterdam, The Netherlands; 3 Department of Pediatrics, Erasmus University Medical Center, Rotterdam, The Netherlands; 4 Center for Lysosomal and Metabolic Diseases, Erasmus University Medical Center, Rotterdam, The Netherlands; 5 Department of Neurology, Neuromuscular Division, University of Rochester Medical Center, University of Rochester, NY, USA

**Keywords:** 3D tissue engineering, skeletal muscle, fibro-adipogenic progenitors, muscle disease, fibrosis, fatty replacement

## Abstract

Human skeletal muscle models often lack important supportive cell types. Here we developed a co-culture three-dimensional tissue engineered skeletal muscle (3D-TESM) model by combining myogenic progenitors (MPs) with genetically-matched immortalized fibro-adipogenic progenitors (iFAPs). FAPs play a crucial physiological role in myogenesis, tissue remodeling and extracellular matrix (ECM) formation. We demonstrate that co-culture 3D-TESMs effectively recapitulate these processes under controlled conditions, thereby enhancing contractile force, muscle tissue integrity and longevity, as well as improving ECM deposition compared to MP-only 3D-TESMs. Moreover, using pro-fibrotic and pro-adipogenic cell culture compositions we were able to mimic pathological features typically observed in muscular dystrophies: excessive ECM production and the formation of fatty infiltrations. This study provides an advanced skeletal muscle model, with enhanced functional and structural properties, capable of recapitulating pathophysiological processes that require FAPs.

## Introduction

Muscular dystrophies are a heterogeneous group of genetic diseases that is characterized by impaired muscle function, often a progressive weakening of contractile force accompanied by pathological changes such as fibrosis and fat replacement.^1–4^ In addition to reduced strength and mobility, patients can experience pain, fatigue, compromised psychosocial well-being and lower overall quality of life.^1,2^ Disease severity and age at symptom onset varies and ranges from slightly reduced mobility to life-threatening cardiac or respiratory complications.^3^ Despite a strong focus on both targeted and supportive therapy development, progress remains limited due to the inadequacies of existing disease models, which frequently fail to mirror the complexity and maturity of human muscle pathology.^5,6^

Current skeletal muscle disease models each present specific limitations. Two-dimensional (2D) muscle cell cultures, while useful for studying molecular mechanisms and biochemical outlines of diseases, lack the three-dimensional (3D) architecture and the complex spatial cell–cell and cell-matrix interactions characteristic of native muscle tissue. Moreover, 2D-cultured myotubes are prone to myotube detachment due to spontaneous contractions, preventing contractile readouts and reaching maturity.^6^ In contrast, small animal models permit contractility measurements and offer a complex, whole-organism context. As such, these models enable research into organ interactions, metabolism, and systemic responses. However, animal models have often been proven to be poor predictors of human responses due to interspecies differences, yielding results with limited translational relevance.^5,7^ Large animal models, which often more closely resemble human physiology than rodent models, are associated with high costs and ethical concerns, making them less ideal for disease modeling.^8–10^ A promising approach to overcoming some of these limitations is the development of human three-dimensional (3D) muscle models.^11,12^ 3D muscle models offer a highly controlled and physiologically relevant environment, using cells of the patient to mimic native tissue organization. They enable the longer-term study of muscle function, including contractility, force generation and cellular interactions, while also allowing for high throughput drug testing. Although these models seem to bridge the gap between *in vitro* studies and *in vivo* physiology, most described 3D models consist of a myocyte monoculture and still lack key supportive resident cell types. These resident cell types are critical for replicating the structural and functional features of human skeletal muscle.^13–15^ This is particularly relevant when studying late-stage disease features, in which prolonged disease development and tissue remodeling are key factors.

One such supportive cell type is the fibro-adipogenic progenitor (FAP), which plays a crucial role in muscle homeostasis and regeneration.^16,17^ FAPs form a population of multipotent mesenchymal progenitor cells residing within the interstitial space of skeletal muscle, capable of differentiating into adipocytes, fibroblasts or osteocytes. Main cell surface marker for the identification of FAPs is platelet-derived growth factor receptor α (PDGFRα).^17^ Under physiological conditions, FAPs transiently expand in response to muscle injury, contributing to extracellular matrix (ECM) production, cytokine secretion, and growth factor release, all of which promote satellite cell activation, differentiation, and fusion.^16^ In healthy conditions FAPs return to a quiescent state, after regeneration. Disruption of this tightly regulated balance leads to pathological FAPs expansion, excessive ECM deposition, and aberrant differentiation into fibroblasts or adipocytes, culminating in the replacement of functional muscle fibers with fibrotic or fatty tissue.^18^ These processes have been implicated in the pathogenesis of multiple skeletal muscle diseases, including Duchenne muscular dystrophy (DMD), limb girdle muscular dystrophy and facioscapulohumeral muscular dystrophy (FSHD).^19–23^

Human skeletal muscle is a heterogeneous tissue composed of myogenic, stromal, vascular, neural and immune cells. These cells interact within specific microenvironments and are regulated by specific signaling cascades. Recapitulating muscle tissue *in vitro* remains challenging due to the need for specific culture conditions, which may not be compatible between cell types. Nonetheless, there has been a surge in 3D model advancements in the past decade, resulting in more complex models that have improved the replication of specific features present in native muscle tissue. These advancements have resulted in co-culture models combining myogenic cells with motor neurons, (myo)fibroblasts, bone or tenocytes.^24–29^ This has demonstrated an improvement in myofiber alignment due to the addition of (myo)fibroblasts and has resulted in platforms that allow to study the development and interplay between different tissues. They all served their purpose in demonstrating the advantages of co-culture versus myogenic monoculture models and they replicated key features *in vitro* previously unattainable. However, FAPs have been absent from current co-culture *in vitro* 3D skeletal muscle models, despite their evident relevance, limiting the ability to study their role in skeletal muscle maturation, tissue integrity and their role in disease development.

In this study, we describe a co-culture 3D tissue engineered skeletal muscle (3D-TESM) model composed of myogenic progenitors (MP) and FAPs. Compared to MP-only 3D-TESMs the co-culture construct resulted in both functional and structural improvements. Moreover, due to the multipotent lineage of FAPs we were able to induce both fibrogenic and adipogenic differentiation in a 3D setting, which demonstrates the potential of FAPs in future muscular dystrophy 3D-models. Overall, these results validate an improved human-relevant 3D-TESM model, highlighting the need for co-cultures in tissue engineered skeletal muscle.

## Results

### Myogenic progenitor and fibroadipogenic progenitor cell populations

**Figure 1.**
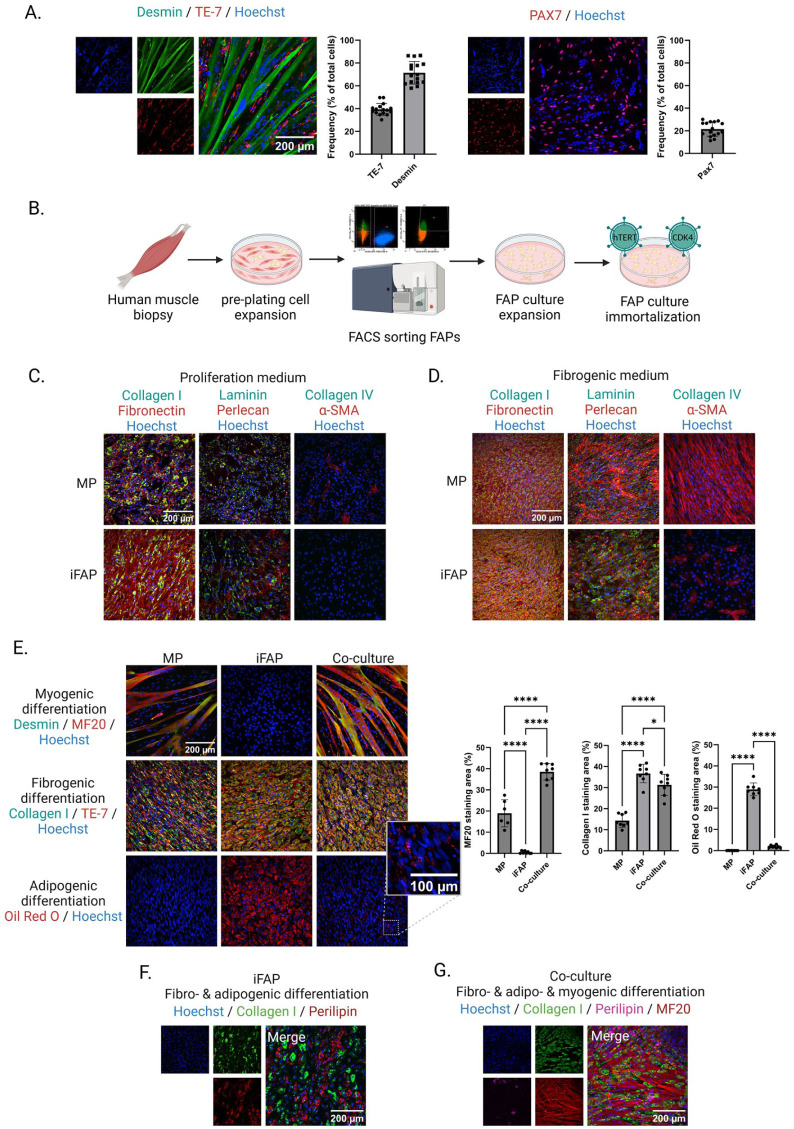
Characterization of MPs, iFAPs and co-cultures in 2D: (a) MP monolayer culture after 3 days of myogenic differentiation, stained for desmin, TE-7 and PAX7 and the associated quantifications (*N* = 16, independent stained wells), (b) graphical overview of FAP isolation, expansion and immortalization. (c) MP versus iFAP monolayer cultures after 2 days of proliferation, with multiple ECM protein immunodetections, (d) MP versus iFAP monolayer cultures after 5 days of fibrogenic differentiation, with multiple ECM protein immunodetections, (e) MP and iFAP monolayer cultures and co-cultures after myo-, fibro- or adipogenic differentiation and their respective immunodetections. Bar graphs depict quantifications of MF20, Collagen I and Oil Red O. Statistical significance was assessed using a one-way ANOVA, followed by Tukey correction for multiple comparisons, *p < 0.05, ****p < 0.0001. Error bars represent standard deviations. *N* = 8, depicting independently stained wells, (f) double lineage differentiation of iFAPs after fibro- and adipogenic media was added, immunodetections with collagen I and perilipin, and (g) triple differentiation of a co-culture, resulting in myotubes, fibroblasts and adipocytes, immunodetections with MF20 (recognizes all myosin heavy chain isoforms), collagen I and perilipin.

Previously described^30,31^ unaffected induced pluripotent stem cells (iPSC)-derived myogenic progenitors (MPs) from two unrelated mosaic FSHD patients were here further characterized by immunofluorescence microscopy. Antibodies binding to desmin, TE-7 and PAX7, to respectively stain myocytes, fibroblasts and satellite cells, were used. This demonstrated that MPs are a heterogeneous population consisting of mostly myogenic cells, fibrogenic cells and satellite cells (Figure 1(a)). Percentages do not add up to 100% due to overlap in staining’s.

Primary fibroadipogenic progenitors (FAPs) were FACS-sorted from skeletal muscle biopsy-derived pre-plating populations and subsequently immortalized (iFAPs) by stable expression of hTERT and CDK4 to allow for extended cell culturing (Figure 1(b)). The iFAPs originated from the same patients as the iPSC-derived MPs, yielding genetically matched MP and iFAP pairs. In this study, we focus on unaffected FAPs and MPs (confirmed by genetic studies, data not shown).

### iFAPs show characteristic multilineage capacity in 2D co-cultures

FAPs are involved in multiple regulatory processes in skeletal muscles. One of their main contributing factors is the production of extracellular matrix (ECM) essential for physical scaffolding and (bio)chemical support of their surroundings.^17,32,33^ As the heterogeneous MP populations consist partially of ECM-producing fibroblasts, we aimed to investigate the difference in ECM producing capacity between iFAPs and MPs. Specifically, we compared ECM production between both cell types, in the presence or absence of TGF-β, which enhances pro-fibrotic signaling (Figure 1(c) and (d)). During cell proliferation, iFAPs produced a clear fibronectin and perlecan network, covering most of the visualized area (Figure 1(c)). In contrast, MPs secreted less ECM overall but did show alpha smooth muscle actin (α-SMA)-positive cells, which may suggest the presence of myofibroblasts within the MP culture.^34,35^ Fibrogenic differentiation (growth medium supplemented with TGF-β), which stimulates the expansion of fibrogenic cells and thereby the production of ECM, resulted in high levels of collagen I, fibronectin, laminin and perlecan, in both MP and iFAP cultures. MPs produced more α-SMA stress fibers than iFAPs after fibrogenic differentiation, in line with the initial α-SMA-positive cells detected during proliferation. No collagen IV was detected in any of the conditions.

Next to their supportive ECM-producing role, FAPs are involved in developing pathological hallmarks in skeletal muscle diseases, such as fibrosis and fatty replacement. FAPs are multipotent and can differentiate into fibroblasts and adipocytes under appropriate conditions. To demonstrate lineage specificity, we performed myo-, fibro- and adipogenic differentiation on MPs, iFAPs and co-cultures (1:1 ratio; Figure 1(e)). Myotube formation after myogenic differentiation was observed in the MP and co-culture conditions, but not in the iFAP-only condition. Collagen I and TE-7 staining was clearly visible in all three cultures after fibrogenic differentiation. Collagen I was most present in iFAP cells, followed by the co-culture condition, both significantly higher than MP-only cells (*p* < 0.001). No adipocytes were formed in the MP-only condition after adipogenic differentiation (0% ORO staining area). However, as expected adipocytes did form in the iFAP cultures (28.9% ORO staining area) and, to a lesser extent, in the co-culture conditions (2% ORO staining area). Double lineage differentiation with fibro- and adipogenic stimuli on an iFAP-only culture demonstrated the multilineage capacity in a single culture. Here, the individual iFAP cells differentiated in fibroblasts and adipocytes with no clear overall preference (Figure 1(f)). Induction of triple differentiation by adding myo-, fibro- and adipogenic stimuli to the media of a co-culture sample, resulted in the co-formation of myotubes, fibroblasts and fat droplets (Figure 1(g)).

Overall, we demonstrated that both MPs and iFAPs can be directed toward their respective lineages within a co-culture. This allows for simulating skeletal muscle disease hallmarks in monolayer cultures (e.g. fibrosis and fat production), previously not attainable with myogenic cell monocultures. To exclude the possibility that these differentiation characteristics were exclusively present in this pair of cell lines, the 2D cell culture characterization was validated in a cell line combination from an independent patient, yielding comparable results (Supplemental Figure 1).

### Increased contractile forces in co-culture 3D-TESMs

**Figure 2.**
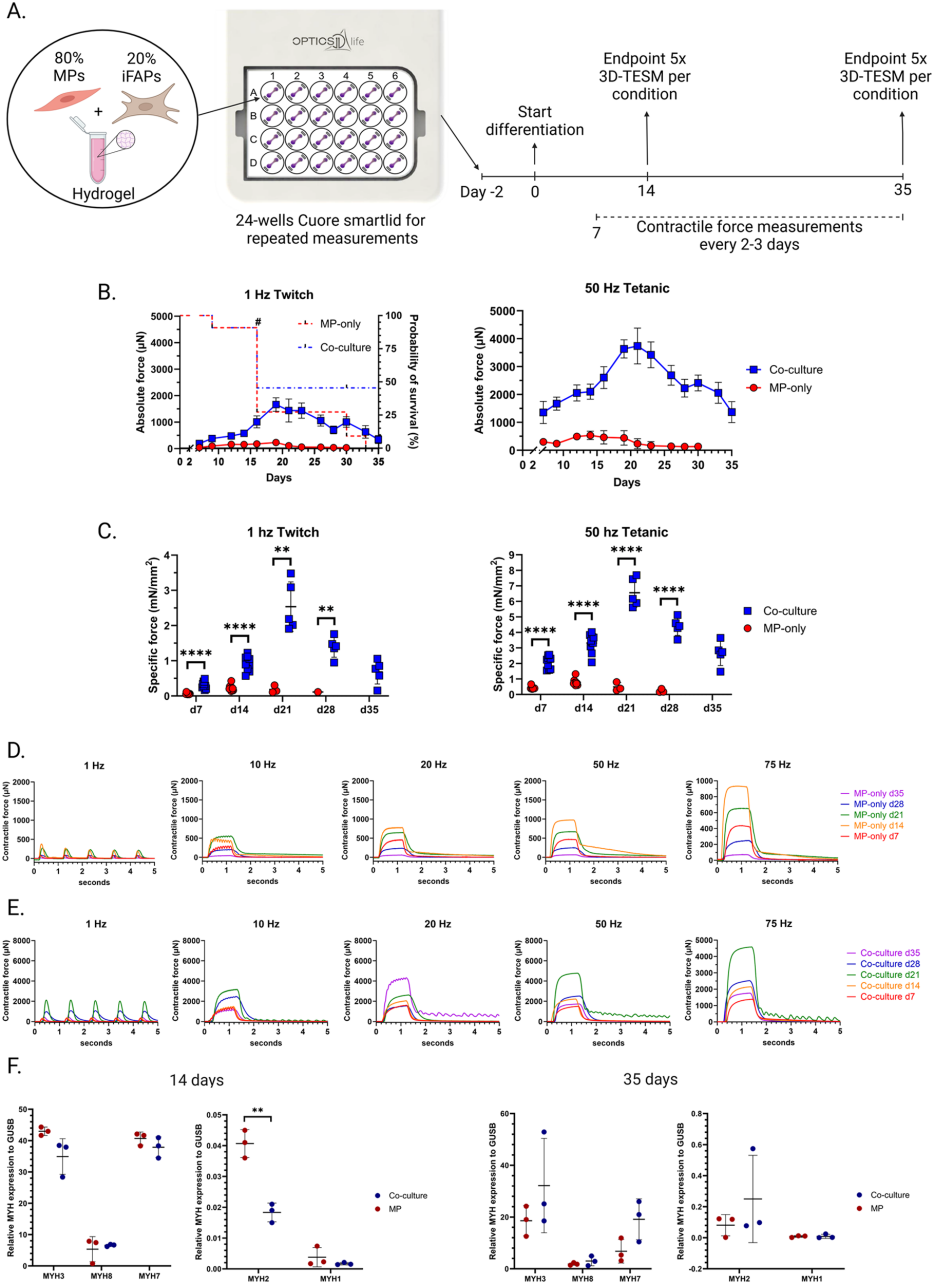
iFAP effect of 3D-TESMs contractility: (a) graphical overview of the experiments conducted with the 24-wells Cuore smartlid platform, (b) absolute contractile force after 1 or 50 Hz EPS over the course of 35 days, comparing MP-only to co-culture 3D-TESMs. 3D-TESM survival plot added to the 1 Hz graph (interrupted line; *N* = 11). # = 5x 3D-TESM per condition collected for post analysis, (c) specific force measurements at d7, d14, d21, d28 and d35 after 1 or 50 Hz EPS. *N* = 3–11, representing biological replicates, (d and e) 1, 10, 20, 50 and 75 Hz EPS demonstrating the difference in contractile response between a representative MP-only 3D-TESM (d) and a co-culture 3D-TESM (e) measured every week, and (f) RT-qPCR gene expression data for myosin heavy chain subtypes at day 14 and day 35 of culturing (*N* = 3, biological replicates). Statistical analysis was performed using a Student’s t-test; Error bars represent standard deviation. **p < 0.01. ****p < 0.0001.

Having established the differentiation capacity of mono- and co-cultures in 2D, we generated 3D-TESMs by encapsulating MPs, with or without iFAPs, in a hydrogel composed of Matrigel, fibrinogen and thrombin. The cell suspension was then seeded into a 24-well plate casting mold, designed for the Cuore platform (Figure 2(a)). This platform consists of stainless-steel cantilevers, one of which is flexible, allowing the formation of 3D-TESMs suspended between these cantilevers and measuring their contractile forces. Due to integrated optic fibers and a carbon electrode plate, this platform enables real-time repeated force measurements with high precision.^36^

First, we established the optimal MP-iFAP ratio for co-culture 3D-TESMs in our setup. To this end, co-culture 3D-TESMs were produced with different ratios of MPs and iFAPs, with iFAPs added as 5%, 10%, 20% or 40% of the total cell count. In our experimental setting, we observed no major macroscopic differences between 3D-TESMs with or without iFAPs, within the first days of muscle bundle formation. Cells cast in hydrogel were cultured in proliferation medium for 2 days before differentiation was initiated, followed by 14 days of culturing in differentiation medium and regular electrical pulse stimulation (EPS) measurements between day 7 and day 14. Tetanic EPS (50 Hz) resulted in an increased contractile force output proportional to the percentage of iFAPs added to the 3D-TESM; the higher the percentage of iFAPs in the co-culture 3D-TESM, the greater the contractile force (Supplemental Figure 2A). This was observed over multiple timepoints in the course of a week (day 7 until day 14 post differentiation). Addition of 40% iFAPs to 3D-TESMs resulted in high variability and a deviating contractile wave with an extended relaxation time at day 7, compared to the other conditions tested (Supplemental Figure 2B). Therefore, although resulting in the highest contractile force, 40% iFAPs was considered too high and the optimal percentage in our platforms was concluded to be 20% iFAPs in co-culture 3D-TESMs. Therefore, 20% iFAPs was used for the remainder of the study.

Next, EPS response to 1 (twitch) and 50 Hz (tetanic) stimulations were assessed over a period of 5 weeks of culturing, comparing MP-only with co-culture 3D-TESMs. Absolute force contractions were measured every 2–3 days, starting at day 7 after initiation of differentiation (Figure 2(b)). Contractile force was higher in co-culture 3D-TESMs starting from day 7 and continued to be consistently higher until termination of the experiment at day 35. Specific force (sF) calculations (absolute contractile force normalized to cross-sectional area) showed significantly increased twitch and tetanic specific forces in co-culture 3D-TESMs compared to MP-only 3D-TESMs at all time points (Figure 2(c)). One out of 11 MP-only and 1 out of 11 co-culture 3D-TESMs lost their contractile ability on day 9. After day 14, five 3D-TESMs per condition were used for gene expression analysis and immunostaining. Between day 14 and day 35, all 5 remaining MP-only 3D-TESMs lost their ability to contract, while all 5 remaining co-culture 3D-TESMs retained contractility until the experiment ended on day 35 (Figure 2(b)).

Once a week, a more extensive comparison between representative 3D-TESMs with or without iFAPs was performed with electrical stimulation frequencies of 1, 10, 20, 50, and 75 Hz. Co-culture 3D-TESMs showed a time-dependent contractile force at all EPS measurements, with the largest increase between day 14 and day 21. MP-only 3D-TESMs demonstrated a peak in contractile force on day 14 and a decline afterward. 50 Hz EPS resulted in the highest tetanic response peak for both conditions (Figure 2(d) and (e)).

To demonstrate the difference in muscle fiber maturation between MP-only and co-culture 3D-TESMs, we analyzed myosin heavy chain (MYH) subtype gene expressions. RT-qPCR analysis of MYH subtypes did not show significant differences between MP-only and co-culture 3D-TESMs at both day 14 and day 35 (Figure 2(f)). However, while co-culture 3D-TESMs contained 80% MPs compared to 100% in MP-only 3D-TESMs upon casting, they expressed comparable levels of MYH isoforms at day 14. This may indicate that iFAPs promote fiber formation or help prevent the degradation of existing fibers. The observed differences in contractile force measurements between MP-only and co-culture 3D-TESMs were validated in an independent second pair of genetically matched MPs and iFAPs (Supplemental Figure 3). Moreover, to determine whether the enhancement in contractile forces of iFAP-containing 3D-TESMs was a general effect of stromal support cells or specific to iFAPs, we compared them to iPSC-derived myofibroblasts (MFBs), another ECM-producing stromal cell type.^37^ MFBs have been described to enhance myogenesis to a greater extent than fibroblasts in 3D-TESMs.^26^ While MFBs and iFAPs produced similar levels of ECM, MFBs produced a higher abundance of α-SMA stress fibers compared to iFAPs (Supplemental Figure 4A). Specific force measurements comparing MP-only, MP-MFB co-culture and MP-iFAP co-culture 3D-TESMs revealed that the increase in specific force was unique to the MP-iFAP co-culture 3D-TESMs, as MP-MFB co-culture 3D-TESMs generated the lowest specific forces across all conditions (Supplemental Figure 4B).

In conclusion, MP-iFAP co-culture 3D-TESMs produced significantly higher contractile forces and remained functional for a longer time than MP-only 3D-TESMs. This demonstrates an effect of iFAPs on myogenesis and suggests a role in tissue integrity.

### Co-culture 3D-TESMs maintain ECM levels over time and develop more dystrophin positive fibers

**Figure 3.**
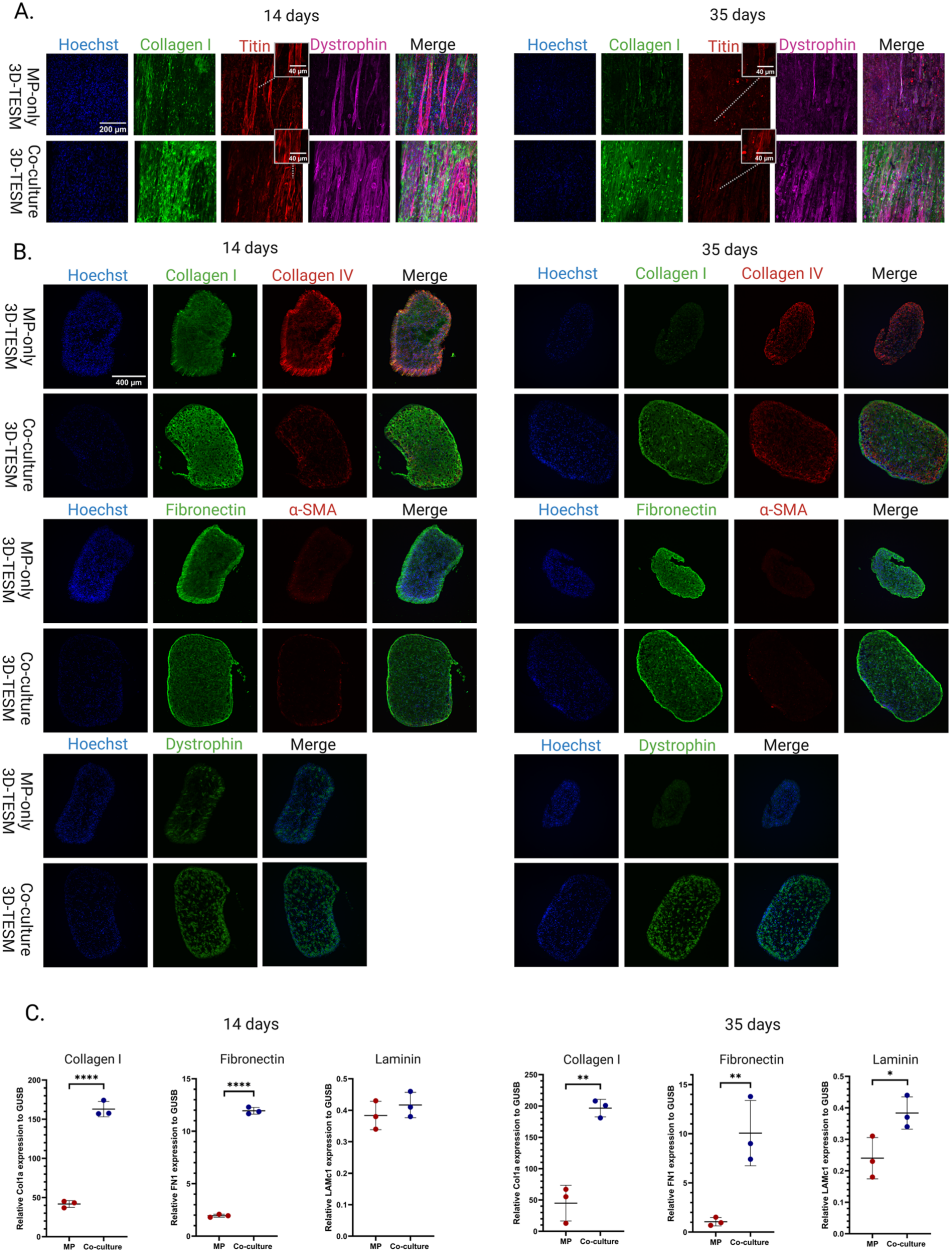
ECM production in 3D-TESMs: (a) 3D-TESM MP-only and co-culture whole mount staining images after 14- and 35-days of culturing comparing titin and dystrophin organization, (b) immunofluorescence staining of different ECM proteins in 14- and 35-day old 3D-TESMs, (c) RT-qPCR quantification of Collagen I, Fibronectin and Laminin in 14- and 35-day old MP-only and co-culture 3D-TESMs (*N* = 3, biological replicates). Statistical analysis was performed using a Student’s *t*-test. Error bars represent standard deviation. *p < 0.05. **p < 0.01. ****p < 0.0001.

As co-culture 3D-TESMs produced stronger contractile forces, the next step was to analyze muscle fiber integrity by immunostaining for titin and dystrophin. Titin is part of the sarcomere and essential for muscle contraction while dystrophin connects the sarcolemma to the actin cytoskeleton and the ECM through the dystrophin-glycoprotein complex, giving structural support.^38,39^ Whole mount staining of 14- and 35-day old 3D-TESMs with and without iFAPs, showed that both conditions had robust sarcomere structures at day 14. However, while these structures persisted in the co-culture condition at day 35, they were largely lost in MP-only 3D-TESMs. These results are consistent with clear differences noted in the dystrophin stainings, where co-culture 3D-TESMs contained more dystrophin positive fibers (Figure 3(a) and (b)).

As FAPs are one of the primary contributors of ECM-production, we wanted to establish their effect on ECM composition in 3D-TESMs by comparing MP-only with co-culture 3D-TESMs on day 14 and day 35 of culturing. On day 14, collagen IV was more present in MP-only 3D-TESMs, but this difference was lost at day 35. In contrast, collagen I was more present in co-culture 3D-TESMs than in MP-only 3D-TESMs on day 14 and day 35. There was no clear difference between the conditions on day 14 for fibronectin, other than seemingly better organized networks in co-culture 3D-TESMs. α-SMA was absent in both 3D-TESMs. Most evident was the loss of ECM components over time in MP-only 3D-TESMs when comparing day 14 to day 35. Co-culture 3D-TESMs seemed to retain their level of ECM-components over time (Figure 3(b)). RT-qPCR analysis of three representative ECM-components (collagen I, fibronectin and laminin) confirmed that co-culture 3D-TESMs produced more ECM and retained these levels of ECM over time, compared to MP-only 3D-TESMs (Figure 3(c)). Overall, these stainings highlight the contribution of iFAPs to ECM production, maintenance and remodeling within a 3D environment.

### Co-culture 3D-TESMs exhibit proteome changes related to tissue remodeling and development

**Figure 4.**
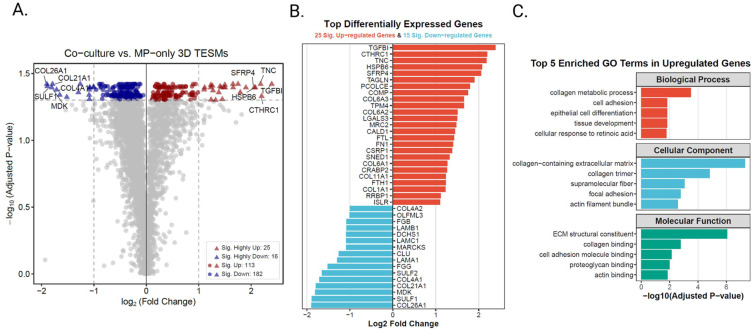
Differential protein expression and functional enrichment analysis between co-culture and MP-only 3D-TESMs: (a) volcano plot showing differentially expressed proteins. Red triangles denote significantly upregulated proteins (log2FC > 2, adjusted *p*-value <0.05, n = 25); blue triangles denote significantly downregulated proteins (log2FC < −2, adjusted *p*-value <0.05, *n* = 16). Red and blue circles represent proteins with moderate fold changes (1 < |log2FC| < 2, adjusted *p*-value <0.05). Gray dots indicate non-significant proteins. Dashed lines mark the significance thresholds (log2FC = ±2, adjusted *p*-value = 0.05), (b) diverging bar plot displaying the 41 significantly differentially expressed proteins (|log2FC| > 2, adjusted *p*-value <0.05). Bars extend from the center axis according to log2 fold change values. Red bars represent upregulated proteins (*n* = 25) and blue bars represent downregulated proteins (*n* = 16) in co-culture 3D TESMs, and (c) gene ontology enrichment analysis of upregulated proteins (log2FC > 1, adjusted *p*-value <0.05, *n* = 138). Top 5 significantly enriched GO terms from Biological Process (BP), Cellular Component (CC), and Molecular Function (MF) categories are shown. No significant GO enrichment was detected for downregulated proteins (not shown). Analysis performed on three MP-only 3D-TESMs and three co-culture 3D-TESMs.

Changes at the proteome level caused by iFAPs were assessed using mass spectrometry-based proteomics by comparing co-culture 3D-TESMs to MP-only 3D-TESMs cultured for 14 days. Proteomic profiling of three MP-only 3D-TESMs and 3 co-culture 3D-TESMs identified 3727 proteins (corresponding to 3727 genes) that were consistently detected across all replicates. Each protein was identified with a minimum of 2 unique peptides per protein, with median protein abundance ranging from 436.3 to 588.7 across samples (Supplemental Table 1). We used a fold change (FC) of > 2 (adjusted *p*-value <0.05) as a cutoff to define differentially expressed proteins, resulting in a total of 41 proteins (Figure 4(a)). Twenty-five proteins were upregulated and 16 proteins were downregulated in co-culture 3D-TESMs compared to MP-only 3D-TESMs (Figure 4(b)). For functional characterization, we performed Gene Ontology (GO) enrichment analysis on differentially expressed proteins. While 25 upregulated and 16 downregulated proteins were identified at a stringent threshold (|log2FC| > 2, adjusted *p*-value <0.05), we adopted a more inclusive cutoff of |log2FC| > 1 (adjusted *p*-value <0.05) for enrichment analysis to better capture changes at the protein level. This is consistent with proteomic studies where fold changes are typically smaller in magnitude compared to transcriptomic data due to post-translational regulation and protein stability. Using these criteria, we identified 138 upregulated and 198 downregulated proteins in co-culture 3D-TESMs. GO enrichment analysis of the upregulated proteins revealed significant functional clustering across all 3 ontology categories: 10 Biological Process (BP) terms, 10 Cellular Component (CC) terms, and 5 Molecular Function (MF) terms (adjusted *p*-value < 0.05; top 5 per category displayed in Figure 4(c)). Interestingly, despite the larger number of downregulated proteins, no significant GO enrichment was observed, suggesting that downregulation occurs in a more scattered, non-pathway-specific manner in response to co-culture conditions. The highly upregulated proteins and their associated GO-terms reveal changes associated with ECM development and remodeling. A combination of GO-terms such as “ECM structural constituent,” “actin binding,” “cell adhesion” and “tissue development” suggests an enhanced ECM formation, tissue-remodeling and differentiated phenotype in co-culture 3D-TESMs compared to MP-only 3D-TESMs. This is likely linked to the activation of iFAPs and their contribution to ECM deposition and remodeling, alongside MP-iFAP crosstalk resulting in enhanced structural integrity. This is in line with higher levels of ECM immunodetections and RNA expression levels as displayed in Figure 3. Upregulated GO-terms related to “cellular response to retinoic acid” suggest a tissue repair response and activation of developmental and morphogen signaling pathways.^40,41^ Overall, these results indicate that co-culture 3D-TESMs undergo more extensive tissue remodeling and developmental processes than MP-only 3D-TESMs.

**Figure 5.**
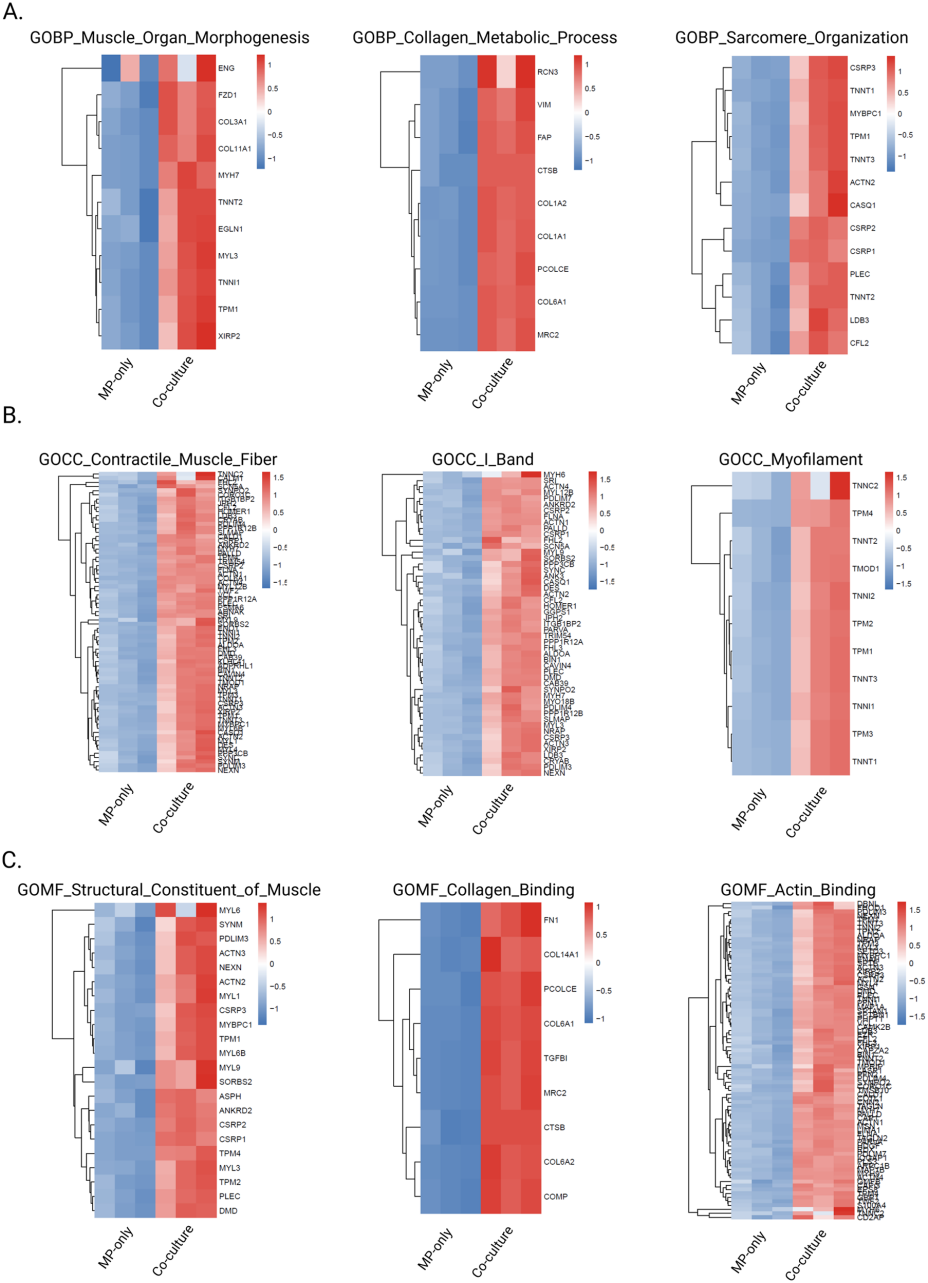
Heatmap visualization of significantly enriched GO BP, CC and MF terms from GSEA: (a–c) heatmaps depicting protein expression patterns for genes within three significantly enriched GO BP (a), GO CC (b) and GO MF (c) terms identified by GSEA (adjusted *p*-value <0.05). Each heatmap shows Z-score normalized abundance values across all six samples (three MP-only 3D-TESMs and three co-culture 3D TESMs). Rows represent individual proteins belonging to each pathway; columns represent individual samples. Color scale indicates Z-score values.

Given the limited number of differentially expressed proteins identified by threshold-based analysis, and the absence of GO enrichment for downregulated proteins, we reasoned that important biological signals might be masked by stringent cutoffs. We therefore performed Gene Set Enrichment Analysis (GSEA), which does not require pre-filtering and instead evaluates whether gene sets show coordinated changes across the entire ranked protein list. All 3727 quantified proteins were ranked by log2 fold change and tested for enrichment against the Hallmark gene set collection (50 canonical pathways) and complete Gene Ontology annotations (BP, CC, and MF). This approach is particularly powerful for detecting subtle but coordinated changes in functionally related proteins. Hallmark GSEA results revealed significant enrichment of pathways associated with ECM remodeling, stromal activation and tissue development, including “epithelial mesenchymal transition” (normalized enrichment score (NES) = 2.70, adjusted *p*-value = 2.3 × 10^−12^), “myogenesis” (NES = 2.48, adjusted *p*-value = 1.66 × 10^−9^), “hypoxia” and “glycolysis” (Supplemental Figure 5). Collectively, these pathways indicate enhanced ECM production, contractile maturation and metabolic adaptation within the co-culture 3D-TESMs, compared to MP-only 3D-TESMs. GSEA using gene ontology categories further highlighted the collective differences between both conditions. A selection of the top hits per GO-category is displayed in Figure 5, showing upregulated pathways in co-culture 3D-TESMs associated with muscle development, structural integrity and ECM-remodeling. The GO biological processes category relevant top hits were: “muscle organ morphogenesis” (NES = 2.40, adjusted *p*-value = 2.92 × 10^−4^), “collagen metabolic process” (NES = 2.34, adjusted *p*-value = 3.62 × 10^−4^) and “sarcomere organization” (NES = 2.24, adjusted *p*-value = 6.44 ×10^−4^). GO cellular component category top 3 hits were: “contractile muscle fiber” (NES = 2.8, adjusted *p*-value = 1.40 × 10^−15^), “I-band” (NES = 2.54, adjusted *p*-value = 4.98 × 10^−9^) and “myofilament” (NES = 2.35, adjusted *p*-value = 6.15 × 10^−5^). Lastly, the top 3 hits for GO molecular function were: “structural constituent of muscle” (NES = 2.55, adjusted *p*-value = 6.1 × 10^−6^), “collagen binding” (NES = 2.43, adjusted *p*-value 7.49 × 10^−5^) and “actin binding” (NES = 2.4, adjusted *p*-value = 2.64 × 10^−10^; Figure 5). An overview of the top 10 up- and downregulated GO-terms within the BP, CC and MF categories are shown in Supplemental Figure 5. Most downregulated GO-terms are related to RNA and/or DNA processes, which may indicate enhanced differentiation in co-culture 3D-TESMs compared to MP-only 3D-TESMs due to enhanced transcriptional silencing. Together, hallmark GSEA and GSEA gene ontology analysis provide evidence that co-culture 3D-TESMs have undergone more muscle tissue- and ECM-remodeling than MP-only 3D-TESMs at day 14 after initiation of differentiation. These results align with the previously described enhanced contractile forces and increased ECM secretion by co-culture 3D-TESMs.

### Morphological improvements in co-culture 3D-TESMs

**Figure 6.**
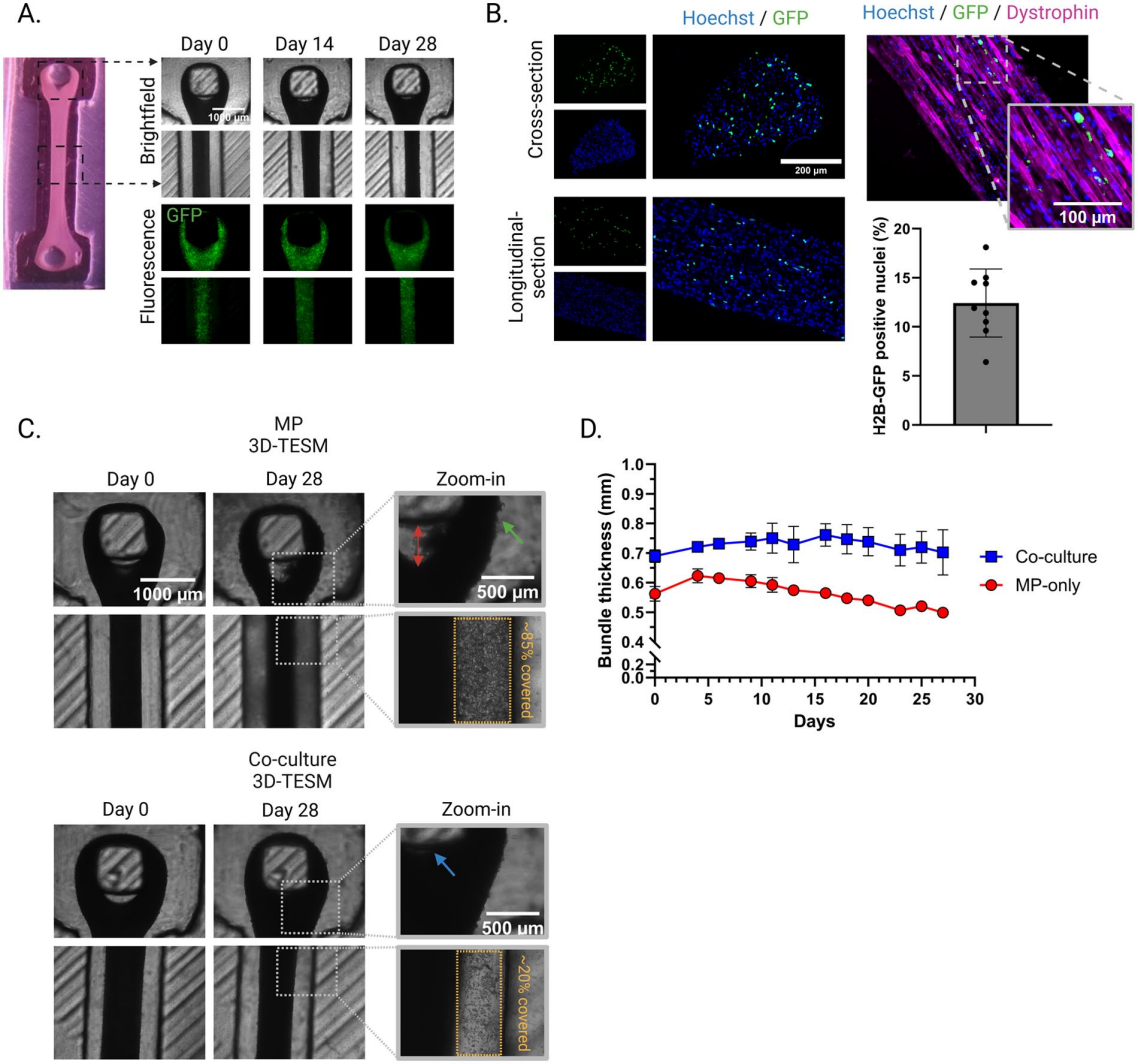
Morphological differences between MP-only and co-culture 3D-TESMs: (a) brightfield and fluorescence images of H2B-GFP-expressing iFAPs in 3D-TESMs at d0, d14 and d28 of culturing, (b) representative cross-section and longitudinal-section images showing the distribution of iFAPs throughout 3D-TESMs, supported by quantification of H2B-GFP-positive nuclei. Each dot represents a cross-section (*N* = 9), coming from three biological replicates. Error bar represents standard deviation. (c) brightfield images demonstrating morphological differences between MP-only and co-culture 3D-TESMs over time. Zoom-in focus on the bottom of the PDMS mold. Red arrow depicts MP-only 3D-TESM detachment from the pillar. Blue arrow shows the attached tightly grown loop of a co-culture 3D-TESM around a PDMS pillar. Green arrow depicts ruffled 3D-TESM surface, and (d) 3D-TESM thickness (mm) over time. Error bar represents standard deviation. *N* = 3, biological replicates.

Having established an enhancement in tissue- and ECM-remodeling due to iFAPs within co-culture 3D-TESMs, we next assessed their effect on 3D-TESM morphology. To this end, H2B-GFP-expressing iFAPs were co-cultured with MPs in 3D-TESMs for 28 days allowing weekly evaluation of their distribution throughout the 3D-TESMs (Figure 6(a)) and analysis after fixation via fluorescence microscopy of cross- and longitudinal sections (Figure 6(b)). For this purpose, 3D-TESMs were cultured in PDMS-molds for convenient image acquisition.^30,31^ The iFAPs were evenly distributed in co-culture 3D-TESMs, with no location-specific accumulation visible. Furthermore, iFAPs grew adjacent to myofibers without fusing into myofibers (Figure 6(b)). Quantification of the number of iFAPs in co-culture 3D-TESMs after 28 days of culturing resulted in a reduction of the total percentage to ~12.5% iFAPs compared to the initial 20% upon casting (Figure 6(b)). This suggests a modest decline in iFAPs and/or an increase of MPs over time.

Structural differences were assessed by brightfield microscopy, focusing on the attachment point of the 3D-TESM to the flexible PDMS pillar and the midsection of the 3D-TESMs. Comparing day 0 to day 28 MP-only 3D-TESMs showed a partial detachment occurring over time between the main body and the loop of the MP-only 3D-TESM (Figure 6(c), red arrow). This detachment did not occur in co-culture 3D-TESMs, where the loop grew tightly around the pillar over time (Figure 6(c), blue arrow). The surface of the MP-only 3D-TESM showed more ruffled edges than the co-culture 3D-TESM (Figure 6(c), green arrow). Furthermore, ~85% of the surface area of the bottom of the PDMS mold was covered with cell debris originating from the MP-only 3D-TESM. The co-culture 3D-TESM showed a ~20% coverage of the surface area. Moreover, analysis of 3D-TESM diameter over time, measured at the midline, showed a diameter decline for MP-only 3D-TESMs while co-culture 3D-TESMs remained a stable thickness (Figure 6(d)). Together, these results show signs of detachment and retraction of muscle fibers in MP-only 3D-TESMs, while co-culture 3D-TESMs seem to maintain tissue integrity.

### iFAP inclusion enables modeling of fibrogenic and adipogenic differentiation in co-culture 3D-TESMs

**Figure 7.**
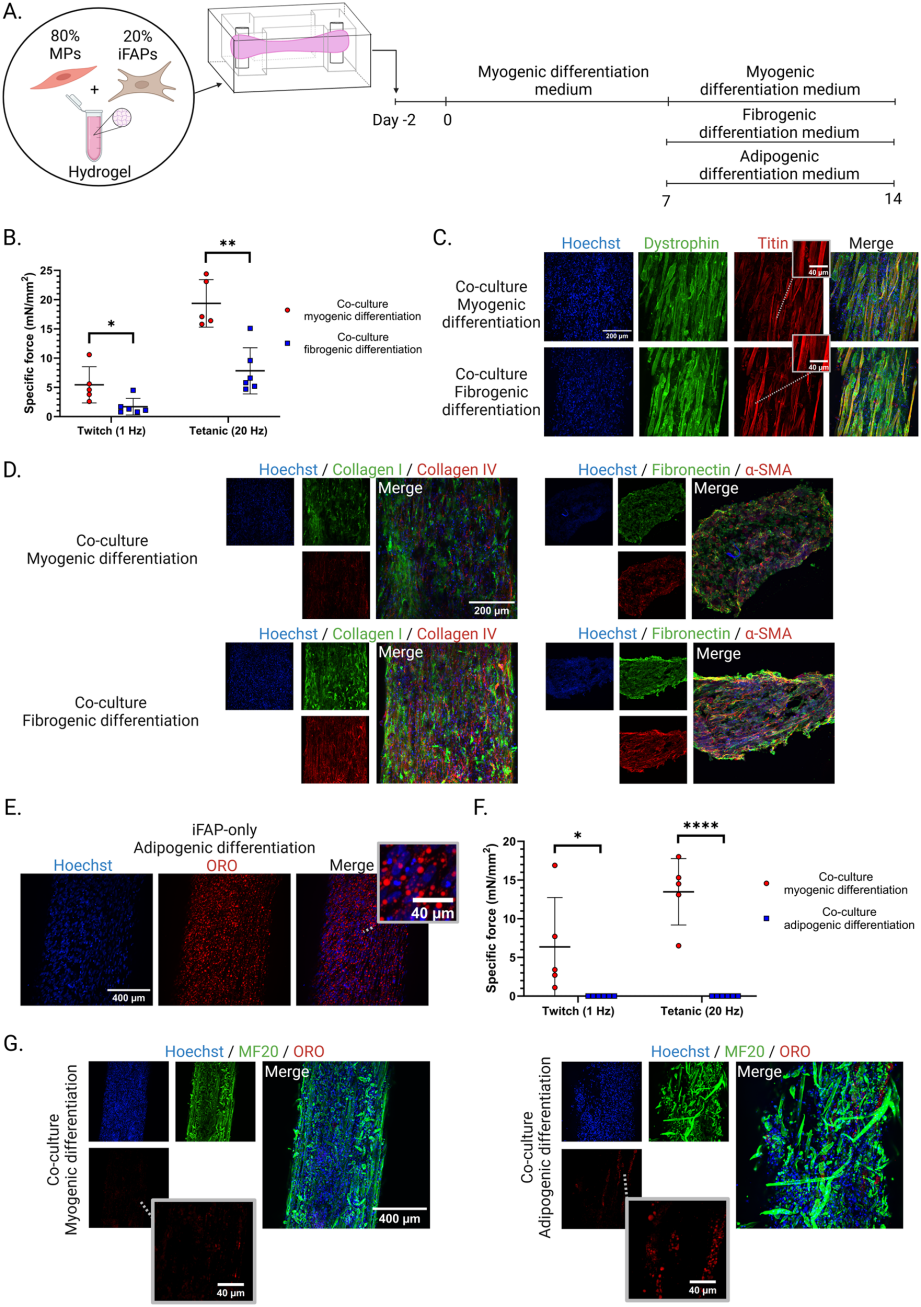
Fibro- and adipogenic differentiation of co-culture 3D-TESMs: (a) experimental setup of myo-, fibro- and adipogenic differentiation cultured in PDMS molds for 14 days, (b) specific forces (mN/mm^2^) of myo- and fibrogenic differentiated co-culture 3D-TESMs after 14 days of culturing, each dot represents a biological replicate, (c) whole mount staining of titin and dystrophin protein, (d) ECM immunofluorescence stainings comparing myo- and fibrogenic differentiation co-culture 3D-TESMs, (e) representative iFAP-only 3D-TESM after adipogenic differentiation, stained for adipocytes with Oil Red O (ORO), (f) specific force (mN/mm^2^) results comparing myo- and adipogenic differentiated co-culture 3D-TESMs at day 14, each dot represents a biological replicate, and (g) representative whole mount staining of myo- and adipogenic differentiated co-culture 3D-TESMs. Statistical analysis was performed using a Student’s *t*-test. Error bars represent standard deviation. *p < 0.05, **p < 0.01, ****p < 0.0001.

In addition to their beneficial physiological roles in skeletal muscle, FAPs are also major contributors to pathological features such as fibrosis and fatty replacement in patients with skeletal muscle diseases. These pathological changes arise from dysregulated and uncontrolled differentiation of FAPs into fibroblasts and adipocytes within an inflammatory environment.^42,43^ To study the differentiation capacity of iFAPs and their potential for disease modeling in a 3D setup, iFAPs were differentiated into fibroblasts or adipocytes in co-culture 3D-TESMs cultured in PDMS molds (Figure 7(a)).

Addition of TGF-β (10 ng/ml) between day 7 and day 14 to the myogenic differentiation medium (fibrogenic differentiation) during 3D-TESM culturing, resulted in a significant decline in specific contractile force (1 Hz, *p* = 0.0255 ; 20 Hz, *p* = 0.0010, Figure 7(b)). Muscle fiber formation did not appear to be affected by fibrogenic differentiation, as indicated by similar dystrophin and titin staining between myogenic and fibrogenic differentiated co-culture 3D-TESMs (Figure 7(c)). However, a difference was evident in their respective ECM secretions. As anticipated, fibrogenic differentiation resulted in increased immunofluorescence staining for collagen I, collagen IV, fibronectin, and α-SMA (Figure 7(d)). This excessive ECM production may have led to stiffening of the 3D-TESMs, which would explain the reduced contractile forces observed in fibrogenic differentiated 3D-TESMs.

Next, iFAPs were differentiated into adipocytes in an iFAP-only 3D-TESM, to demonstrated their adipogenic capacity in a 3D environment (Figure 7(e)). This was followed by adipogenic differentiation of co-culture 3D-TESMs, resulting in a complete loss of contractile force (Figure 7(f)). Furthermore, adipogenic differentiation resulted in the formation of fat droplets in co-culture 3D-TESMs (Figure 7(g)). These fat droplets formed a chain of adipocytes in a pattern resembling a muscle fiber, suggesting that fat had replaced a previously existing fiber or formed along the length of myofibers.^44^ MF20 staining of myotubes revealed that adipogenic differentiation resulted in damaged muscle fibers, explaining the loss of contractile force. This suggests that pro-adipogenic compounds negatively affect muscle fiber maintenance. Taken together, these results demonstrate the multipotency of iFAPs in a 3D setting and their relevance for studying disease-related characteristics under controlled conditions.

## Discussion

The absence of reliable predictive models for human muscle diseases currently hampers our understanding of the pathophysiology of various skeletal muscle disorders and slows the discovery of effective therapies. Skeletal muscle contains a variety of mononuclear cells, including satellite cells, FAPs, adipocytes and immune cells. 3D-TESMs often contain only myogenic cells and therefore lack many resident cell types that are essential for accurately recapitulating physiological and pathological processes *in vitro*. In this study, we incorporated iFAPs into 3D-TESMs and by that recapitulated key physiological functions of these stromal cells in a controlled environment, including ECM deposition, preservation of muscle mass, and promotion of myogenesis. We identified specific force differences between MP-only and co-culture 3D-TESMs over prolonged culturing periods, which was met with a higher probability of survival of co-culture 3D-TESMs. Gene expression and immunofluorescence analysis revealed increased levels of ECM-components present in co-culture 3D-TESMs. Upregulation of processes involved with myogenesis, ECM-remodeling and tissue integrity were confirmed by proteome analysis. Furthermore, morphological analysis showed a stable thickness and less cell/fiber detachment over time in co-culture 3D-TESMs, compared to MP-only 3D-TESMs. Lastly, we demonstrated that iFAPs within a co-culture 3D-TESM were capable of differentiating into fibrogenic or adipogenic cells. While previous *in vivo* studies^15,17,45–47^ have highlighted the supportive role of FAPs and their involvement in pathophysiological hallmarks, our work extends these observations by showing that these effects can be reenacted within a controlled engineered muscle construct. This work establishes a foundation for developing more physiologically relevant tissue culture models of disease, thereby enabling the identification and evaluation of targeted therapeutic interventions.

The characterization of monolayer MPs, iFAPs and co-cultures resulted in expected cell type-specific lineages. We showed that previously generated iPSC-derived MPs^31^ are heterogeneous and consist of myoblasts, satellite cells and (myo)fibroblasts, in agreement with other iPSC-derived MP populations showing mixed compositions.^48^ Upon fibrogenic differentiation, MP cultures developed α-SMA–positive stress fibers, in line with previously described fibroblast-to-myofibroblast transition in response to TGF-β signaling.^49,50^ In agreement with Contreras et al.,^51^ we also observed iFAP-to-MFB differentiation upon TGF-β treatment, although to a lesser extent than in the MP-only cultures. This may reflect the closer functional proximity of fibroblasts to MFBs compared to FAPs.^52,53^ Importantly, by supplementing medium with specific pro-fibrogenic or pro-adipogenic compounds, iFAPs were able to be directed toward desired lineages, demonstrating the utility of MP-iFAP co-culturing for controlled lineage specification. Moreover, iFAPs differentiated into both fibroblasts and adipocytes within a single culture dish, showing their capacity in multilineage differentiation. Finally, co-cultures of iFAPs and MPs were capable of forming myotubes, fibroblasts and adipocytes simultaneously, highlighting the interplay between MPs and FAPs. Together, these findings underscore the versatility and potential of MP–iFAP co-cultures as a platform for studying lineage plasticity and cell–cell interactions between multiple cell types.

FAPs are known to facilitate myogenesis and enhance the differentiation of myogenic cells.^15,46,54^ FAPs produce trophic factors, such as interleukin-6, that promote muscle growth and during injury FAPs are known to induce satellite cell proliferation, resulting in regeneration of muscle tissue.^17,18,55–58^ In this study, we investigated the effects of FAPs on contractile force in co-culture 3D-TESMs. Contractile force is a hallmark of functional, mature, and healthy muscle tissue. An increase in contractile force in tissue engineered muscle would suggest an improved myogenic quality and physiological relevance.^30,59,60^ 3D-TESMs with and without iFAPs were cultured for a total of 35 days. Co-culture 3D-TESMs exerted higher contractile forces than MP-only 3D-TESMs, starting from the first day of measurements (day 7). Quantitative specific force measurements were significantly different at all time points, for both twitch (1 Hz) and tetanic (50 Hz) contractions. The biggest significant specific force difference occurred at day 28, showing *a* ± 20 times higher tetanic specific force in co-culture 3D-TESMs compared to MP-only 3D-TESMs (5.9 vs 0.3 mN/mm^2^, *p* < 0.0001, *N* = 5). Both MP-only and co-culture representative 3D-TESMs showed an electrical stimulation frequency dependent contractile trace, with a maximum sustained force at 50 Hz. A review by Vesga-Castro et al.^61^ compared specific force measurements reported across 40 studies, each applying different cell lines, culturing times and/or *in vitro* muscle models. The specific force values of these muscle constructs were between 0.06 and 50 mN/mm^2^. Our co-culture 3D-TESM reached a peak specific force of 6.6 mN/mm^2^ at day 21 in the Cuore Smartlid system. Within the PDMS mold system, our co-culture 3D-TESMs reached a peak force of 19.4 mN/mm^2^ at day 14. This highlights the difficulty of directly comparing results between different systems and studies, which is further complicated due to different culturing times per study before conducting contractile force measurements. Yet, these results do show that our co-culture construct falls within the higher range of contractile forces produced by 3D-TESMs currently described. Regardless, specific force values are still lower than the ones reported in human adult native muscle, with specific force values up to 84 mN/mm^2^.^62^ Therefore, there remains a need for continued advancements. Optimizing hydrogel and medium composition, adjusting the shape of attachment points and local tissue geometry may all, to some extent, influence contractile force output and are worth investigating in the future.^30,61,63–65^

Next to improved functional properties, adding iFAPs to 3D-TESMs resulted in improved structural properties, regarding ECM-production and morphology. Co-culture 3D-TESMs produced more collagen I, fibronectin and dystrophin than MP-only 3D-TESMs. Moreover, the levels of these ECM proteins stayed relatively stable over time, as no clear differences were found between day 14 and day 35. This did not apply to the MP-only 3D-TESMs, which were met with a decrease in ECM-components over time. The higher levels of ECM in co-culture 3D-TESMs could partially explain the previously described higher levels of contractile force, as it is known that ECM supports myofibers and facilitates the transmission of force.^66^ Previous studies have described a direct correlation between ECM remodeling and enhanced contractility, highlighting the role of proper ECM-production in tissue engineered muscle.^59,67–69^ We further described improved morphology in co-culture 3D-TESMs, as visible by a smooth surface of the 3D-TESMs and less cell debris, compared to MP-only 3D-TESMs. Moreover, 3D-TESM thickness, as measured by the diameter at the midline, remained stable in co-culture 3D-TESMs compared to a decrease over time in MP-only 3D-TESMs. Another clear difference was seen in the number of dystrophin-positive muscle fibers. Dystrophin is described as the shock absorber of muscle tissue, with additional roles in structural support and mechanotransduction.^39^ Furthermore, it directly links the actin cytoskeleton to the ECM. The importance of dystrophin is evident from DMD patients, in whom the absence of dystrophin results in a severe, progressive muscle-wasting disease.^70^ The higher levels of dystrophin and ECM-components in co-culture 3D-TESMs may therefore explain the stability of the construct over time, with less loss of contractile force, ECM and thickness over time compared to MP-only 3D-TESMs. This was supported by proteome analysis as demonstrated by an upregulation of GSEA GO-terms involved with ECM-remodeling, muscle development and tissue integrity in co-culture 3D-TESMs (e.g. “ECM structural constituent,” “muscle organ morphogenesis,” “actin binding” and “sarcomere organization”).

FAPs are considered the main fibrogenic cell in skeletal muscle tissue.^54,71^ Our characterization of iFAPs in mono- and co-cultures confirms their intrinsic fibrogenic capacity and provides valuable mechanistic insights into ECM-production and remodeling. This study demonstrates that iFAPs secrete high levels of ECM under basal conditions and respond to pro-fibrotic stimuli, such as TGF-β, by increasing their ECM production. This increased ECM deposition correlates with reduced contractile forces, suggesting tissue stiffness. These findings align with previous reports demonstrating that FAP-mediated fibrosis results in stiffness and that TGF-β signaling drives myofibroblast differentiation of FAPs.^33,51^ Consistently, addition of TGF-β to both 2D and 3D co-cultures enhanced key ECM-component expression, including collagen I, fibronectin and α-SMA. The emergence of α-SMA-positive stress fibers, characteristic of myofibroblasts, underscores the successful recapitulation of a fibrotic response *in vitro*, as increased numbers of myofibroblasts have been linked to muscular diseases.^72,73^ Overall, this demonstrates the utility of the co-culture 3D-TESM model to study pathological ECM remodeling and fibrosis.

In addition to their role in fibrosis, FAPs are key regulators of fatty replacement in skeletal muscle following injury. Under healthy conditions, FAPs are quiescent and contribute to muscle homeostasis. However, in response to various stimuli such as chronic inflammation, mechanical stress, or metabolic dysregulation, FAPs can undergo differentiation into adipocytes, leading to the accumulation of ectopic fat within muscle tissue.^74,75^ Previous studies have demonstrated the adipogenic potential of FAPs *in vitro*, including differentiation within hydrogel-mediated 3D constructs, highlighting their ability to produce mature adipocytes in a controlled setting.^4,42,76,77^ Consistently, our iFAPs differentiated into adipocytes in monoculture 3D-TESMs as well, confirming their intrinsic adipogenic capacity. Importantly, we report the differentiation of iFAPs into adipocytes within a co-culture 3D-TESM, alongside myogenic progenitors. This multilineage differentiation not only recapitulates aspects of the *in vivo* muscle microenvironment but also adds functional and structural complexity to the engineered tissue, emphasizing the potential of FAP-MP co-cultures as a model to study cellular crosstalk, tissue remodeling, and pathological fat infiltration in skeletal muscle. Here, we induced adipogenic differentiation by adding pro-adipogenic compounds to the differentiation medium, which in turn also inhibits muscle regeneration.^78^ Ideally, future research should look into endogenous adipogenic differentiation upon inflicted mechanical damage using diseased FAP cell lines. Together, co-culture 3D-TESMs allows for studying FAP-MP cross-talk in the development of adipocytes as seen in multiple muscular diseases.

3D muscle models consisting of myogenic and stromal cell co-cultures have been described previously, often describing an optimal ratio of 1:1.^26,79,80^ Our MP cells consist of a heterogeneous cell population, containing a mix of myogenic and stromal cells at an approximate ratio of 3:2. When adding 20% iFAPs to the total cell count, the ratio myogenic to stromal cells shifts to 1:1, aligning with earlier established preferred ratios for *in vitro* 3D tissue engineered muscle.

A limitation of the current study is the lack of additional supportive cell types, such as immune cells and endothelial cells that can facilitate vascularization. These would allow for better uptake and clearance of small molecules and testing of drugs in the future. Another improvement to the system would be applying repeated EPS as “training”, which could further enhance functional survival and maturation, as demonstrated in recent studies.^81,82^ The use of disease affected FAPs in future studies might help underscore the role of FAPs in pathological process, without the need for pro-fibrotic and pro-adipogenic compounds to force differentiation.

In summary, we enhanced functional and structural properties of 3D-TESMs by combining MPs and iFAPs into a co-culture construct. This model successfully recapitulates both the physiological and pathological hallmarks FAPs are involved in within a controlled 3D environment. This allows for studying the role of FAPs in both health and disease. Due to the wide range of improvements and possibilities for disease modeling, we believe that co-culturing MPs with FAPs will form the new benchmark for physiologically relevant skeletal muscle modeling. Moreover, this model offers a robust platform for studying muscle pathophysiology and therapeutic interventions in the future.

## Materials and methods

### Myogenic progenitor culture (MP)

Myogenic progenitors (MPs) were previously generated by myogenic differentiation of hiPSC.^31,83^ For 2D culturing, MPs were seeded on dishes coated with ECM (1:200 diluted, Sigma-Aldrich) and expanded in growth medium (GM) consisting of DMEM High Glucose (Gibco, Waltham, MA), 10% Fetal Bovine Serum (FBS), 1% Penicillin-Streptomycin (P/S, Gibco) and 100 ng/ml bFGF2 (Preprotech, Rocky Hill, NJ). Trypsinization was done by adding 1:1 diluted TrypLE express reagent (Gibco) in PBS (Gibco). Differentiation of MPs into myotubes was induced with differentiation medium (DM) consisting of DMEM High Glucose, 1% P/S, 1% Insulin-Transferrin-Selenium (ITS-X, Gibco) and 1% KnockOut Serum Replacement (KOSR, Gibco).

### Fibroadipogenic progenitor culture (FAP)

FAPs were isolated and immortalized by adapting methods previously described.^20,84^ In short, frozen cells from muscle biopsies were provided by the FSHD Research Center at University of Rochester Medicine. The cells were expanded in GM and then trypsinized, washed and stained for 30 min on ice with antibodies: PDGFRa-PE (BD Pharmingen, 556002) and CD56-APC (BD Pharmingen, 555518). The labeled cells were filtered through a 40-µm cell strainer, pelleted and re-suspended in medium consisting of DMEM-F12 (Gibco), 1% ITS-X and 1% P/S for isolation by flow cytometer. Cells were sorted on a FACSAria III (BD Biosciences) sorter using the FACSDiva software. Double cells were excluded. Compensations were adjusted based on single stained controls. PDGFRa+/CD56- fraction was defined as FAPs. FlowJo software (FlowJo LLC, Ashland, OR, USA) was used for data analysis and data elaboration. 2D culturing of FAPs was performed on ECM-coated dishes and expanded in FAP-medium (FM) consisting of Ham’s F10 nut mix High Glucose (Gibco), 20% FBS, 1% P/S, 10 mM HEPES (Gibco), 1 µM dexamethasone (Sigma, D4902) and 2.5 ng/ml bFGF2.

Immortalization of human FAPs was performed by transduction with a retroviral vector containing a sequence encoding the catalytic subunit of human telomerase reverse transcriptase (hTERT) and human cyclin-dependent kinase 4 (Cdk4) in the presence of 4 µg/ml polybrene. Positive selection markers for transduction were neomycin (0.3 mg/ml) and hygromycin (0.2 μg/ml), respectively.^85^

### Myofibroblast culture (MFB)

Myofibroblasts were generated following a myogenic differentiation of hiPSC protocol previously published.^86^ Cells were sorted on a FACSARIA III sorter using the FACSDiva software. Double cells were excluded. Compensations were adjusted based on single stained controls. PDGFRa+/CD56+ fraction was defined as MFBs. FlowJo software was used for data analysis and data elaboration. 2D culturing of MFBs was performed on ECM-coated dishes and cells were expanded in FM.

### Fibrogenic and adipogenic differentiation medium

Fibrogenic differentiation was induced by supplementing medium with 10 ng/ml TGF-β (Gibco). Adipogenic differentiation was induced by supplementing medium with adipogenic induction medium (AIM) components, consisting of 10 µg/ml Insulin (Roche), 0.5 mM 3-isobutyl-L-methylxanthine (IBMX, Sigma), 1 µM Dexamethasone (Sigma) and 5 µM Rosaglitazone (Sigma). In 2D, 3 days of GM combined with AIM was followed by switching to adipogenic maintenance medium (AMM) up to a total of 14 days, consisting of GM supplemented with 10 ug/mL Insulin. For 3D-TESM adipogenic differentiation, 3D-TESMs were differentiated for 7 days in 3D DM followed by 7 days of culturing with 3D DM supplemented with AIM-components. Combinations of fibro-, adipo-, and/or myogenic differentiation was achieved by supplementing GM or DM with TGF-β and/or AIM-components for 5 days.

### Generation of 3D tissue engineered skeletal muscles on PDMS molds

Polydimethylsiloxane (PDMS) molds were fabricated using a direct peeling method as previously described.^87^ Summarized, using a Ultimaker 2 + FDM printer (Ultimaker, Utrecht, the Netherlands) T-bone-shaped chambers on a circular negative mold made of acrylonitrile butadiene styrene (ABS; Ultimaker) were produced. Uncured PDMS was prepared by mixing prepolymer and curing agent at a 10:1 (w/w) ratio (DOW Corning, MI), degassed, and poured over the printed molds. After a second degassing step removing residual air bubbles, PDMS was cured at 75°C for 2 h. Once cured, PDMS molds were removed from the ABS negative molds. Individual chambers (50 µl) containing two cylindrical pillars (1 mm diameter, 3.2 mm height) were cut out and affixed to a 24-well plate (CELLSTAR; Greiner Bio-One, Alphen aan den Rijn, the Netherlands), using uncured PDMS as adhesive, followed by 24 h curing at room temperature. Before seeding, PDMS molds were sterilized by immersion in 70% ethanol for 15 min, washed with PBS, and subsequently exposed to UV for 15 min. Finally, PDMS molds were incubated overnight in 1% Pluronic F-127 (Sigma-Aldrich, Amsterdam, the Netherlands) to prevent nonspecific cell binding.

A hydrogel mixture was made consisting of bovine fibrinogen (5% v/v; Sigma-Aldrich, Amsterdam, the Netherlands) dissolved in DMEM high glucose (final concentration 2 mg/ml), Matrigel growth factor reduced (20% v/v; Corning Life Sciences, NY, United States), thrombin from human plasma (1.6% v/v; Sigma-Aldrich, Amsterdam, the Netherlands) dissolved in 0.1% BSA in PBS (0.8 U/ml final concentration) and GM without Streptomycin (73.4% v/v). All components were incubated, defrosted and kept on ice for the duration of the experiment. MPs and iFAPs were detached using TrypLE reagent (1:1 diluted in PBSl; Life Technologies, Carlsbad, CA, United States) and suspended in GM (600,000 cells/mold). Next, MPs + iFAPs were first mixed with fibrinogen and Matrigel, after which thrombin was quickly added and the cell-hydrogel mixture was pipetted into the PDMS chamber. To allow for polymerization of the mixture the 24-wells plate harboring the PDMS chambers was incubated at 37°C and 5% CO2 for 30 min. Afterward, GM without streptomycin and supplemented with 6-aminocaproic acid (1.5 mg/ml final concentration; Sigma-Aldrich, Amsterdam, the Netherlands) and TGF-β inhibitor (10 μM; Selleck Chemicals, Cologne, Germany) as added. After 48 h, GM was replaced with 3D differentiation medium (3D DM), composed of DMEM high glucose supplemented with 1% ITS-X (Gibco, Waltham, MA, United States), 1% knock-out serum replacement (Gibco, Waltham, MA, United States), 1% penicillin-G (Sigma-Aldrich, Amsterdam, the Netherlands) and 6-aminocarproic acid (2 mg/ml; Sigma-Aldrich, Amsterdam, the Netherlands). 3D-TESMs were maintained under constant agitation at 65 rpm (Thermo Fisher Scientific, Landsmeer, the Netherlands) at 37°C and 5% CO2, with full medium volume replacements every 2–3 days.

Independent 3D-TESMs were generated from cell lines of a single patient for the main figures. Each construct was established and cultured separately, representing within-donor biological replicates. Supplemental Figures 1–3 present 3D-TESMs generated from cell lines from a second, independent patient.

### Electrical stimulation and force measurements of 3D-TESMs cast on PDMS molds

Contractile function of 3D-TESMs was assessed using electrical pulse stimulations using an Arduino Uno Rev3 equipped with an Adafruit motorshield V2 with supplied software (both Adafruit, New York, NY, United States). 3D-TESMs were subjected to a single twitch pulse (1 Hz), followed by a tetanic pulse (20 Hz). During stimulations, one of the pillars displacement was captured at 60 frames per second using Thorlabs Microscope filer cubes controlled with Thorlabs imaging software (Thorlabs, Dortmund, Germany). Analysis of the contractions was performed by ImageJ Fiji software or with a custom-modified Python script. Brightfield images of each 3D-TESM were acquired at 4x magnification using the EVOS FL Imaging System (Invitrogen Thermo Fisher Scientific, Landsmeer, the Netherlands), to measure the height of attachment points at the pillar. Absolute forces were calculated with the following formula: Force (Newton) = (Ewt3)/(2a2(3L–a)) * δ with a PDMS stiffness of 1.59 ± 0.2792 MPa. Specific forces were calculated by dividing the absolute forces by the cross-sectional area (CSA), which were deduced from measuring the width at the midline of 3D-TESMs and assuming an elliptical shape with a width/height ratio of 0.6 (For specifications see Supplemental Figure 6). During culturing we could only measure the width of bundles, therefore this formula was necessary to calculate the CSA.

### Generation of 3D-TESMs using Cuore smartlid

Experiments were executed with Cuore Smartlid V2.0 (Optics11-Life, Amsterdam, the Netherlands), consisting of a custom-made 24-wells casting mold, cantilever plate, stimulation plate, fiber plate, base frame and Smartlid seal. All components were sterilized by washing with 70% ethanol (EtOH) for 15 min, followed by washing with PBS and placed under UV-light for 15 min. Lastly, the components were placed in an autoclavable bag and sterilized using an autoclave cycle at 121°C and 15 psi for 15 min. Next the culture chambers on the casting mold were incubated overnight with 1% Pluronic F-127. Similar to culturing in PDMS molds, 600,000 cells per well were combined with hydrogel and thrombin and subsequently pipetted into the culture chambers (50 µl volume). Immediately after casting the cell-hydrogel mix, the Smartlid was assembled by placing the casting mold in the base frame, followed by adding the fiber plate and cantilever plate. The cantilever plate harbors 24-pairs of cantilevers, one stiff and one flexible, distancing 4 mm from each other. The flexible cantilever is coupled with an optical fiber sensor, allowing for measuring cantilever displacement upon electrical stimulations. Assembly of the Smartlid was followed by incubating at 37°C and 5% CO2 for 30 min to polymerize the hydrogel, after which 3D GM supplemented with 6-aminocaproic acid and TGF-β inhibitor was added. After 48 h medium was replaced by 3D DM. Full medium volume was refreshed every 2–3 days.

### Electrical stimulation and force measurements of 3D-TESMs cast on cuore Smartlid

To perform electrical stimulations, a stimulation plate was added to the Smartlid unit starting day 7 of differentiation. The stimulation plate consists of 24-pairs of carbon plate electrodes, which are in line with the 3D-TESM suspended between the two cantilevers. The stimulation plate was connected to an external pulse generator (Optics11-Life). Standard force measurements were performed with 5 V, 10 ms pulse amplitude and 1 or 50 Hz frequencies. Frequencies ranging from 1 to 75 Hz were utilized to assess differences in contractile response curves between conditions. Twitch contractions were calculated by taking the average of five consecutive 1 Hz pulses. Tetanic contractions were calculated by taking the average of five consecutive 50 Hz pulses, with 30 s intervals in between. The stimulation plate was kept in distilled sterile water in between every time point measurement and UV-sterilized for 15 min before use.

Via the fiber plate, an infrared broadband laser source was directed through fiber optics toward the flexible cantilevers. A DeltaSens interferometer system (Optics11-Life) was used to track interference patterns over time, allowing for simultaneous measurements of multiple 3D-TESMs. Calculating contractile forces upon deflection of cantilevers using optic fibers with the Cuore Smartlid are published in detail by Iuliano et al.^36^ Cuore software and Cuore viewer V2.3 (Optics11-Life) were used to visualize and analyze electrical signal amplitudes. Specific forces were calculated by dividing absolute forces by the CSA. CSA was deduced from width measurements, assuming an elliptical shape and a width/height ratio of 1.75, which was specific for 3D-TESMs cultured on the Smartlid (For specifications see Supplemental Figure 6).

### RNA isolation, cDNA synthesis, and RT-qPCR

After contractile force measurements, 3D-TESMs were detached from the PDMS pillars or Smartlid cantilevers, snap-frozen in liquid nitrogen, and subsequently stored at −80°C. Next, RNA was isolated using the miRNeasy mini kit including a DNase step according to the manufacturer’s instructions (Qiagen, Venlo, the Netherlands), followed by cDNA synthesis of equal amounts of RNA using the RevertAid™ H minus First Strand cDNA Synthesis Kit (Thermo Fisher Scientific, Landsmeer, the Netherlands). For RT-qPCR analysis, cDNA was diluted 10x in RNase-free water, mixed with 5 μl SybrGreen IQ-supermix (Bio-Rad, Veenendaal, the Netherlands) and 10 pmol of both forward and reverse primers, and analyzed on the CFX 96 or CFX 384 machine (Bio-Rad, Veenendaal, the Netherlands). Primer sequences can be found in Table 1.

**Table 1.** List of primer sequences used in this study.

Target	Forward primer sequence (5′ à 3′)	Reverse primer sequence (5′ à 3′)
GUSB	CTCATTTGGAATTTTGCCGATT	CCGAGTGAAGATCCCCTTTTTA
COL1A	GCCAGACTATCCCCTTCCTC	GGGTGACTCTGAGCCGTC
FN1	CCCATCAGCAGGAACACCTT	GTGGGAGCATCCAGTTTGGT
LAMC1	GAGGCAAGATATCGCCGTGA	TGCCCAAGAACTTTGCAGGA
MYH1	CTCCTCTTTGTTGGGGCAAC	CAGCTTATTCAAATTCTCCC
MYH2	TAAAAAGCTCCAAGAACTGT	TGCGCTCCCTTTCAGACTTT
MYH3	CTTGTGGGCGGAGGTCTG	AGCAGCTATGCCGAACACTT
MYH7	CTGTCCAAGTTCCGCAAGGT	TCATTCAAGCCCTTCGTGCC
MYH8	ATTTCCACCAAGAACCCA	AAAGGATTCTGCCTCTGG

### Immunofluorescence staining

2D cultures or 3D-TESMs were fixed with 4% paraformaldehyde (PFA; Sigma-Aldrich, Amsterdam, the Netherlands), respectively, for 10 min or 1 h at room temperature, washed with PBS for three times, and stored at 4°C in PBS. For staining of 2D myotubes, fixed cells were permeabilized in 0.1% Triton-X (Sigma-Aldrich) in PBS for 10 min, washed with PBS, and blocked in blocking buffer (3% BSA and 0.1% Tween-20 in PBS) for 30 min. Next, cells were washed once with PBS and incubated with primary antibodies in 0.1% BSA (Sigma-Aldrich) and 0.1% Tween-20 in PBS for 1 h at room temperature. Cells were then washed with 0.1% Tween-20 in PBS and with PBS for 2 min each and incubated with secondary antibodies and Hoechst diluted in 0.1% BSA and 0.1% Tween-20 in PBS for 30 min at room temperature in the dark. Finally, cells were washed once with 0.1% Tween-20 in PBS and once with PBS for 2 min each and stored in PBS at 4°C before imaging.

For whole mount immunostaining, fixed 3D-TESMs were blocked and permeabilized in 0.3% Triton-X, 3% BSA, and 0.1% Tween-20 in PBS, on agitation (180 rpm) for 1 h at room temperature. 3D-TESMs were then washed once with PBS and incubated with primary antibodies diluted in 0.1% Triton-X, 0.1% BSA, and 0.1% Tween-20 in PBS for 1 h at room temperature. Next, 3D-TESMs were washed once in 0.1% Tween-20 in PBS and once with PBS for 2 min each, after which 3D-TESMs were incubated with secondary antibodies and Hoechst nuclear staining for 30 min at room temperature in the dark. After incubation, 3D-TESMs were washed with 0.1% Tween-20 in PBS and stored in PBS at 4°C before imaging. Cells and 3D-TESMs were imaged using the Andor Spinning disk confocal microscope Dragonfly 200 (Oxford Instruments, Oxford, United Kingdom). Primary and secondary antibodies used in this study are listed in Table 2.

**Table 2.** Antibodies used in this study.

Target	Host	Dilution	Company	Cat. number
αSMA	Mouse	1:1000	Abcam	Ab7817
Collagen I	Goat	1:400	Southern Biotech	1310-01
Collagen IV	Rabbit	1:50	Invitrogen	14-9871-82
Desmin	Rabbit	1:500	Millipore Sigma	04-585
Dystrophin	Rabbit	1:100	Abcam	Ab15277
Fibronectin	Rabbit	1:100	Abcam	Ab2413
Laminin	Rabbit	1:500	Invitrogen	PA1-16730
MF20	Mouse	1:250	DSHB	MF20
PAX7	Mouse	1:50	DSHB	PAX7
Perilipin	Rabbit	1:200	Invitrogen	PA5-72921
Perlecan	Rat	1:20	Invitrogen	MA5-14641
Titin	Mouse	1:50	DSHB	9D10-S
TE-7	Rabbit	1:300	Santa Cruz Biotechnology	sc-73603
Anti-goat-488	Donkey	1:500	Invitrogen	A11055
Anti-mouse-594	Donkey	1:500	Invitrogen	A21203
Anti-rabbit-488	Donkey	1:500	Invitrogen	A21206
Anti-rabbit-594	Donkey	1:500	Invitrogen	A11037
Anti-rabbit-680	Donkey	1:500	LI-COR	926-68073
Anti-rat-594	Goat	1:500	Life Technologies	A21113
Hoechst 33342		1:2000	Invitrogen	H1399

### Oil Red O (ORO) staining

An Oil Red O (ORO) stock solution was prepared by dissolving 3 mg/ml ORO (Sigma-Aldrich) in 100% isopropanol (JT Baker, Avantor, Radnor, PA, USA), followed by thorough mixing. After 20 min, the ORO stock solution was diluted 3:2 with Milli-Q to prepare an ORO working solution, which was filter sterilized after 10 min. Fixed cells were incubated with isopropanol (60% v/v in MQ) for 5 min, after which the ORO working solution was added. After 10–20 min, cells or 3D-TESMs were washed 2–5 times with PBS until excess stain was no longer observed. Finally, cells were stored in PBS at 4°C until imaging.

### Tissue sectioning of 3D-TESMs

After being fixed, 3D-TESMs were cut in half, removed from the mold, and incubated in 30% sucrose solution (Sigma-Aldrich, Amsterdam, the Netherlands) in PBS for 24 h at 4°C. 3D-TESMs were then embedded in O.C.T.-filled (Avantor, VWR, Amsterdam, the Netherlands) plastic cryomolds (Tissue-Tek, Sakura, CA, United States), snap frozen by submersion in a bath of chilled 2-methylbutane (Sigma-Aldrich, Amsterdam, the Netherlands) in liquid nitrogen, and stored at −80°C until sectioning. Slices of 20 μm thick were cut from 3D-TESMs using the cryostat Leica 3050S (Leica Biosystems, Deer Park, IL, United States) chilled at −20°C. Glass slides with cryosection slices were stored at −20°C until staining.

For immunostaining, glass slides were removed from −20°C and incubated for 24 h at room temperature. Next, cryosections were rehydrated two times in PBS for 30 min at room temperature. Glass slides were boiled in Tris-EDTA (10 mM Tris, 1.25 mM EDTA; pH 9) buffer for 15 min for antigen-retrieval, washed once with PBS for 5 min at room temperature, and then blocked in blocking solution (5% milk powder (Sigma-Aldrich, Amsterdam, the Netherlands) in 0.05% Tween-20 in PBS) for 10 min at room temperature. Primary antibodies were diluted in blocking solution, added to the sections, and the sections were incubated overnight at 4°C. After incubation, glass slides were washed three times in 0.05% Tween-20 in PBS for 10 min and subsequently incubated with secondary antibodies and Hoechst (1:2000, Invitrogen) diluted in blocking solution for 1 h in the dark at room temperature. Finally, sections were washed with PBS, dried, mounted with a cover slide using Prolong antifade mountant (Thermo Fisher Scientific, Landsmeer, the Netherlands), and stored at 4°C before being imaged using the Andor Spinning disk confocal microscope Dragonfly 200 (Oxford Instruments, Oxford, United Kingdom).

### Proteomics data analysis

For proteome analysis, MP-only and co-culture 3D-TESMs were generated on PDMS-molds and cultured for 14 days. Both conditions were prepared in biological triplicates. At day 14, 3D-TESMs were washed with PBS and snap frozen with liquid nitrogen. Before downstream processing 3D-TESMs were lysed in sodium dodecyl sulfate lysis buffer and protein concentrations were measured.

Proteins were digested into peptides using trypsin, after which peptides were dissolved in water/formic acid (100/0.1, v/v). Peptide samples were analyzed by online C18 nano–liquid chromatography coupled to tandem mass spectrometry (nanoLC–MS/MS) using an Ultimate 3000 nano-gradient HPLC system (Thermo Fisher Scientific, Bremen, Germany) connected to an Exploris 480 mass spectrometer (Thermo Fisher Scientific). Samples were first loaded onto a cartridge precolumn (300 µm × 5 mm, C18 PepMap, 5 µm, 100 A) and subsequently separated on a homemade analytical nanoLC column (50 cm × 75 µm; Reprosil-Pur C18-AQ, 1.9 µm, 120 A; Dr. Maisch, Ammerbuch, Germany). Peptides were eluted using a linear gradient from 2% to 40% solvent B (20/80/0.1 water/acetonitrile/formic acid, v/v) in 120 min at a flow rate of 250 nL/min. The nanoLC column was drawn to a ~10 µm tip and served as the electrospray emitter.

Mass spectrometry was performed in data-independent acquisition (DIA) mode using higher-energy collision dissociation (HCD). A lock mass of m/z 445.12003 was used for internal calibration. MS1 scans were acquired in the Orbitrap at a resolution of 120,000 over a scan range of 350–1100 m/z with normalized automatic gain control (AGC). MS2 scans were acquired at a resolution of 15,000 using predefined isolation windows (~11 Th wide), ensuring comprehensive and reproducible peptide fragmentation across samples.

### Proteomics data processing

Mass spectrometry raw data were processed using Proteome Discoverer software (version 2.5, Thermo Fisher Scientific). Peptide identification was performed by searching against the UniProt human reference proteome database. Search parameters included: trypsin as the digestion enzyme with a maximum of two missed cleavages; precursor mass tolerance of 10 ppm; fragment mass tolerance of 0.02 Da; carbamidomethylation of cysteine as a fixed modification; oxidation of methionine and N-terminal acetylation as variable modifications. Peptide-spectrum matches (PSMs) were validated using Percolator with a false discovery rate (FDR) threshold of 1% at both peptide and protein levels. Proteins were considered confidently identified if supported by at least two unique peptides. Protein abundance was quantified using label-free quantification method, for example, intensity-based absolute quantification (iBAQ) or precursor ion intensity. After quality control filtering, proteins detected across all six samples (three MP-only 3D-TESMs and three co-culture 3D-TESMs) were retained, resulting in a final dataset of 3727 proteins for downstream analysis.

### Gene ontology enrichment analysis

Gene Ontology (GO) enrichment analysis was performed using the clusterProfiler package (version 4.16.0) in R (version 4.5.0). Differentially expressed proteins were defined as those with log2 fold change > 1 (upregulated) or < –1 (downregulated) and adjusted *p*-value <0.05. Separate enrichment analyses were conducted for upregulated (*n* = 138) and downregulated (*n* = 198) protein sets. The enrichGO function was applied with the following parameters: organism database org.Hs.eg.db (version 3.21.0); ontology categories “Biological Process” (BP), “Cellular Component” (CC), and “Molecular Function” (MF) analyzed separately; universe parameter set to all 3727 quantified proteins to account for detection bias; statistical significance assessed using hypergeometric test with Benjamini-Hochberg correction for multiple testing; significance threshold of adjusted *p*-value <0.05. For visualization, the top 5 enriched GO terms from each category were displayed as bar plots using ggplot2 (version 2.4.0).

### Gene set enrichment analysis (GSEA)

To identify coordinated pathway-level changes, Gene Set Enrichment Analysis (GSEA) was performed using the fgsea package (version 1.34.2) in R. Protein abundance ratios (co-culture vs MP-only 3D-TESMs) exported from Proteome Discoverer were log2-transformed, and all 3727 quantified proteins were ranked by their log2 fold change values. GSEA was conducted against two gene set collections from the Molecular Signatures Database (MSigDB v25.1.1): (1) Hallmark gene sets (*n* = 50 well-defined biological states and processes), and (2) Gene Ontology gene sets encompassing all Biological Process (*n* = 7583), Cellular Component (*n* = 1042), and Molecular Function (*n* = 1855) terms. Gene sets were filtered to include only those with 10–500 genes. Enrichment analysis was performed using the fgsea function. Gene sets with adjusted *p*-value <0.05 were considered significantly enriched.

Normalized Enrichment Scores (NES) were calculated to account for gene set size and correlations between gene sets, with positive NES indicating enrichment in upregulated proteins and negative NES indicating enrichment in downregulated proteins. For visualization of significantly enriched Hallmark pathways, protein abundance values for all detected pathway members were extracted and Z-score normalized across samples. Heatmaps were generated using the pheatmap package (version 1.0.13).

### Statistical analysis

Statistical analysis was performed using GraphPad Prism software version 9.3.1 (GraphPad Prism Inc., La Jolla, CA, USA). Statistical differences between two groups were determined by unpaired Student’s *t*-test analysis. Statistical differences between more than two groups were determined by one-way ANOVA followed by Tukey correction for multiple comparison. All values are shown as mean ± standard deviation (SD) with significance defined as *p* ⩽ 0.05.

## Supplemental Material

sj-docx-1-tej-10.1177_20417314261441552 – Supplemental material for Fibro-adipogenic progenitors enhance functional and structural properties of human 3D tissue engineered skeletal muscles Supplemental material, sj-docx-1-tej-10.1177_20417314261441552 for Fibro-adipogenic progenitors enhance functional and structural properties of human 3D tissue engineered skeletal muscles by Roy Augustinus, Lotte A. de Ridder, Dongxu Zheng, Marnix Franken, Judit Balog, Patrick J. van der Vliet, Alessandro Iuliano, Remko Goossens, Johanna I. Hamel, W. W. M. Pim Pijnappel, Jessica C. de Greef and Silvère M. van der Maarel in Journal of Tissue Engineering
