# Supplementary material for: Fibro-adipogenic progenitors enhance functional and structural properties of human 3D tissue engineered skeletal muscles
Source: J Tissue Eng. 2026 Apr 25;17:20417314261441552. doi: 10.1177/20417314261441552 (PMC13129286; doi:10.1177/20417314261441552)
Supplement: sj-docx-1-tej-10.1177_20417314261441552 – Supplemental material for Fibro-adipogenic progenitors enhance functional and structural properties of human 3D tissue engineered skeletal muscles [file sj-docx-1-tej-10.1177_20417314261441552.docx]

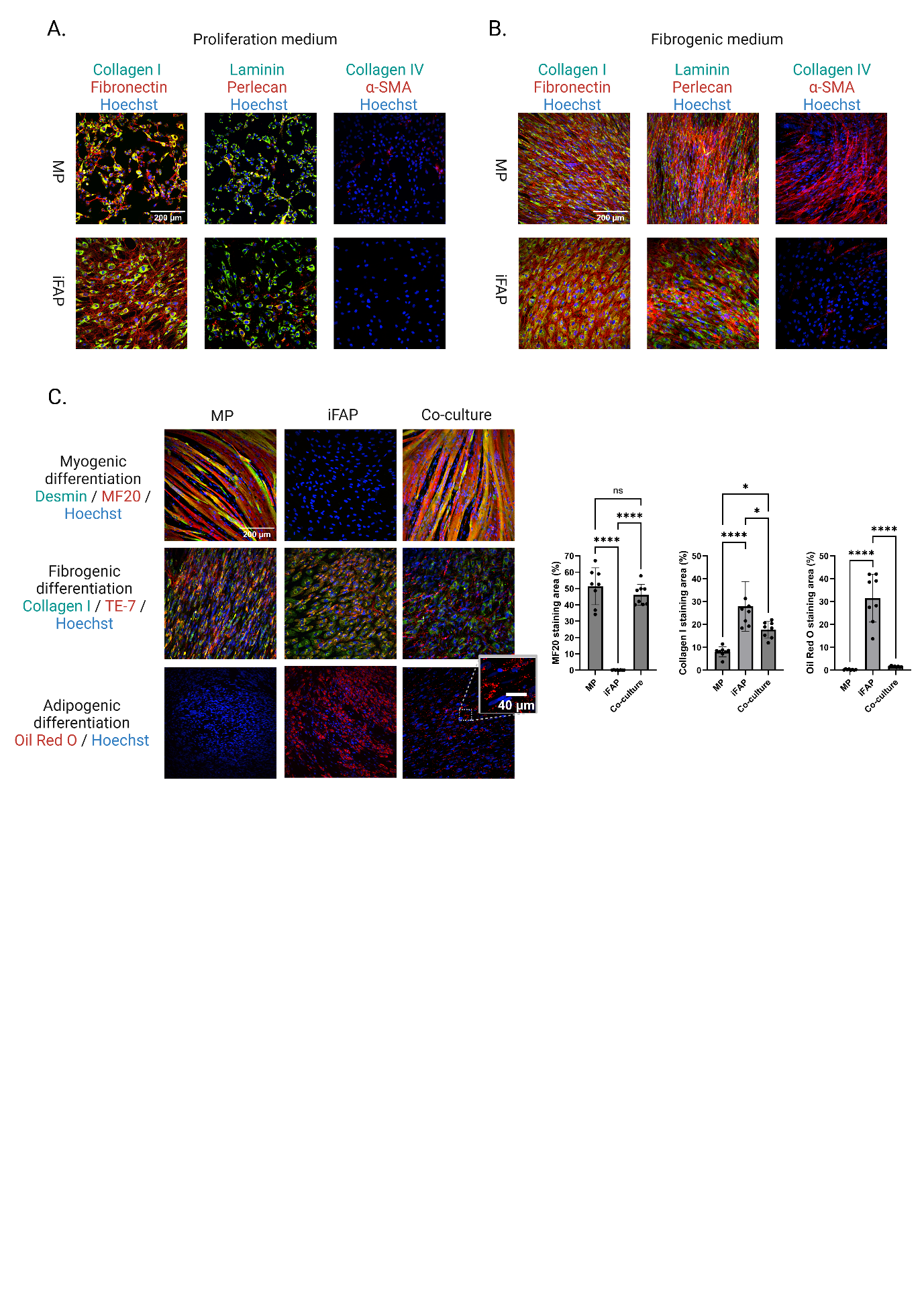


**Supplemental Figure 1. Characterization of MPs, iFAPs and co-cultures in 2D from a second independent patient.** A) MP versus iFAP monolayer cultures after 2 days of proliferation, with multiple ECM protein immunodetections. B) MP versus iFAP monolayer cultures after 5 days of fibrogenic differentiation, with multiple ECM protein immunodetections. C) MP and iFAP monolayer cultures and co-cultures after myo-, fibro- or adipogenic differentiation and their respective immunodetections. Bar graphs demonstrate quantifications for MF20, Collagen I and Oil Red O stainings. Statistical significance was assessed using a one-way ANOVA, followed by Tukey correction for multiple comparisons, *p<0.05, ****p<0.0001. Error bars represent standard deviations. N=8, independently stained wells.


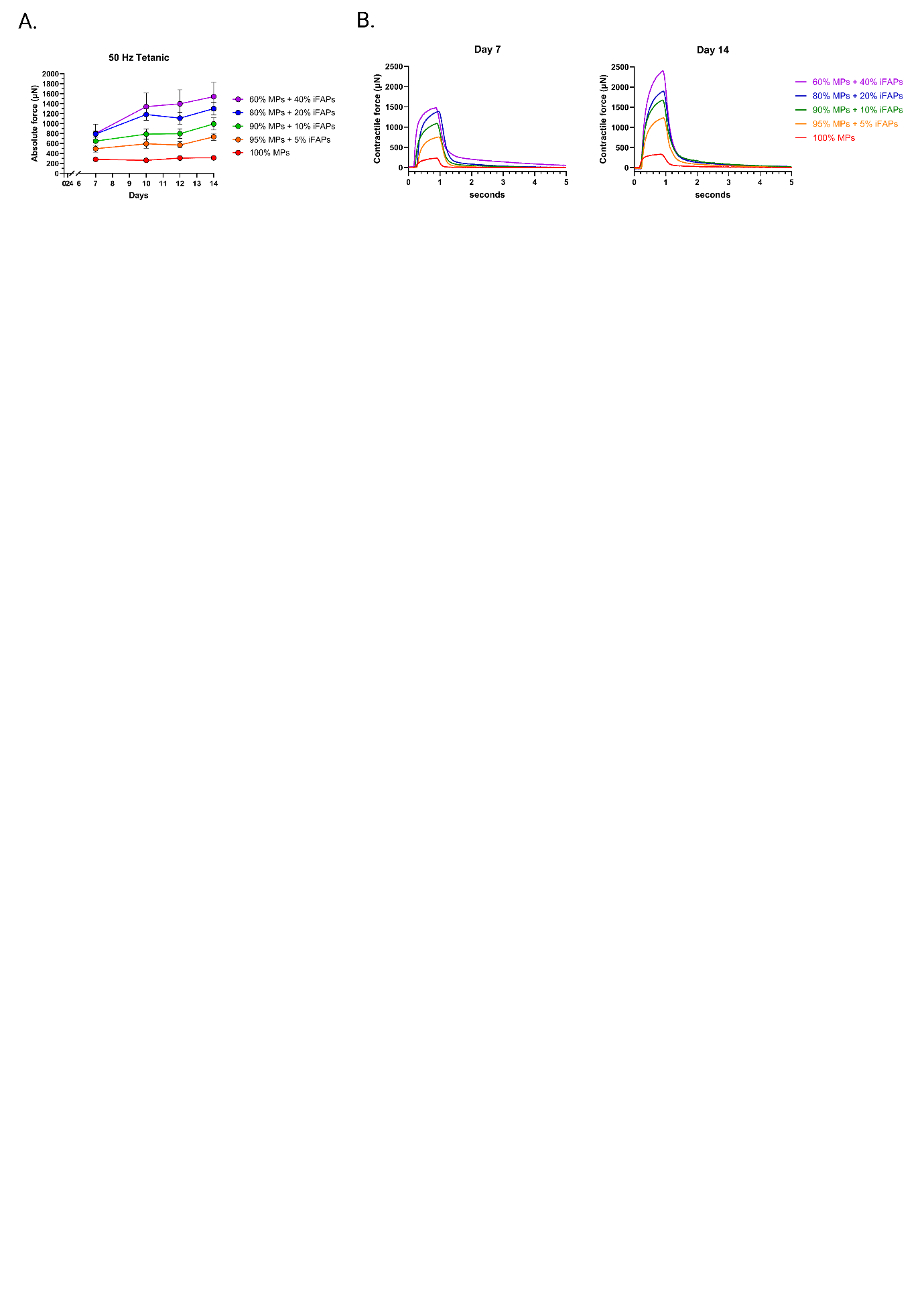


**Supplemental Figure 2. Contractile force results of 3D-TESMs with different percentages of iFAPs .** A) Absolute contractile force after 50Hz EPS between day 7 and 14 of culturing, comparing co-culture 3D-TESMs with different percentages of iFAPs. B) 50 Hz electrical pulse stimulation demonstrating the difference in contractile response between representative co-culture 3D-TESMs at day 7 and day 14. Error bars represent standard deviation.


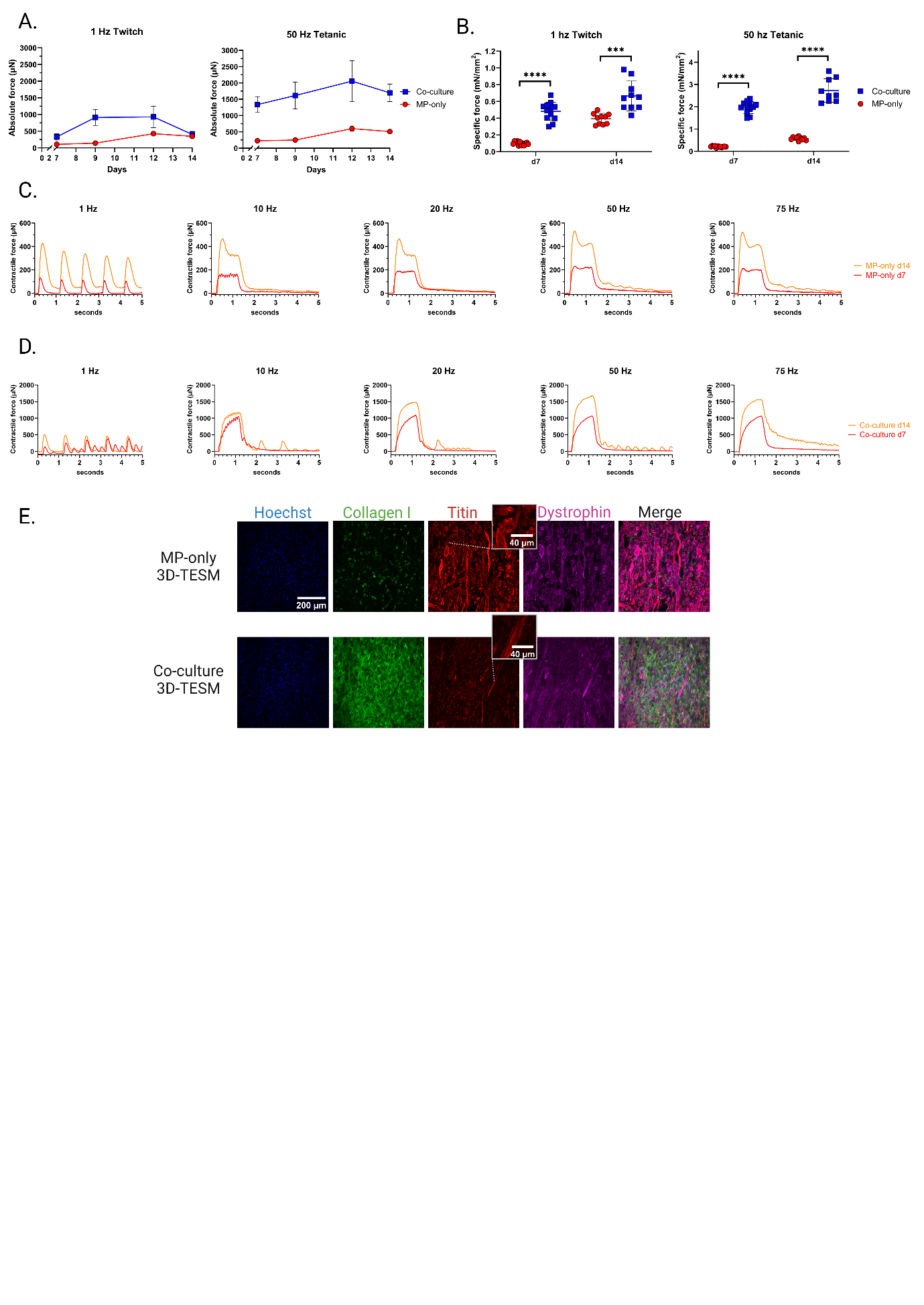


**Supplemental Figure 3. iFAP effect of 3D-TESMs contractility in independent patient-derived cell lines.** A) Absolute contractile force after 1 Hz or 50 Hz EPS over the course of 14 days, comparing MP-only to co-culture 3D-TESMs. B) Specific force measurements at d7 (N=12) and d14 (N=10) after 1 Hz or 50 Hz EPS. Dots represent independent TESMs (biological replicates). C, D) 1, 10, 20, 50 and 75 Hz EPS demonstrating the difference in contractile response between a representative MP-only 3D-TESM and a co-culture 3D-TESM measured at day 7 (C) and 14 (D). E) Whole mount stainings for collagen I, titin and dystrophin at day 14 of culturing. Statistical analysis was performed using a Student's t-test. ***p<0.001, ****p<0.0001. Error bars represent standard deviation.


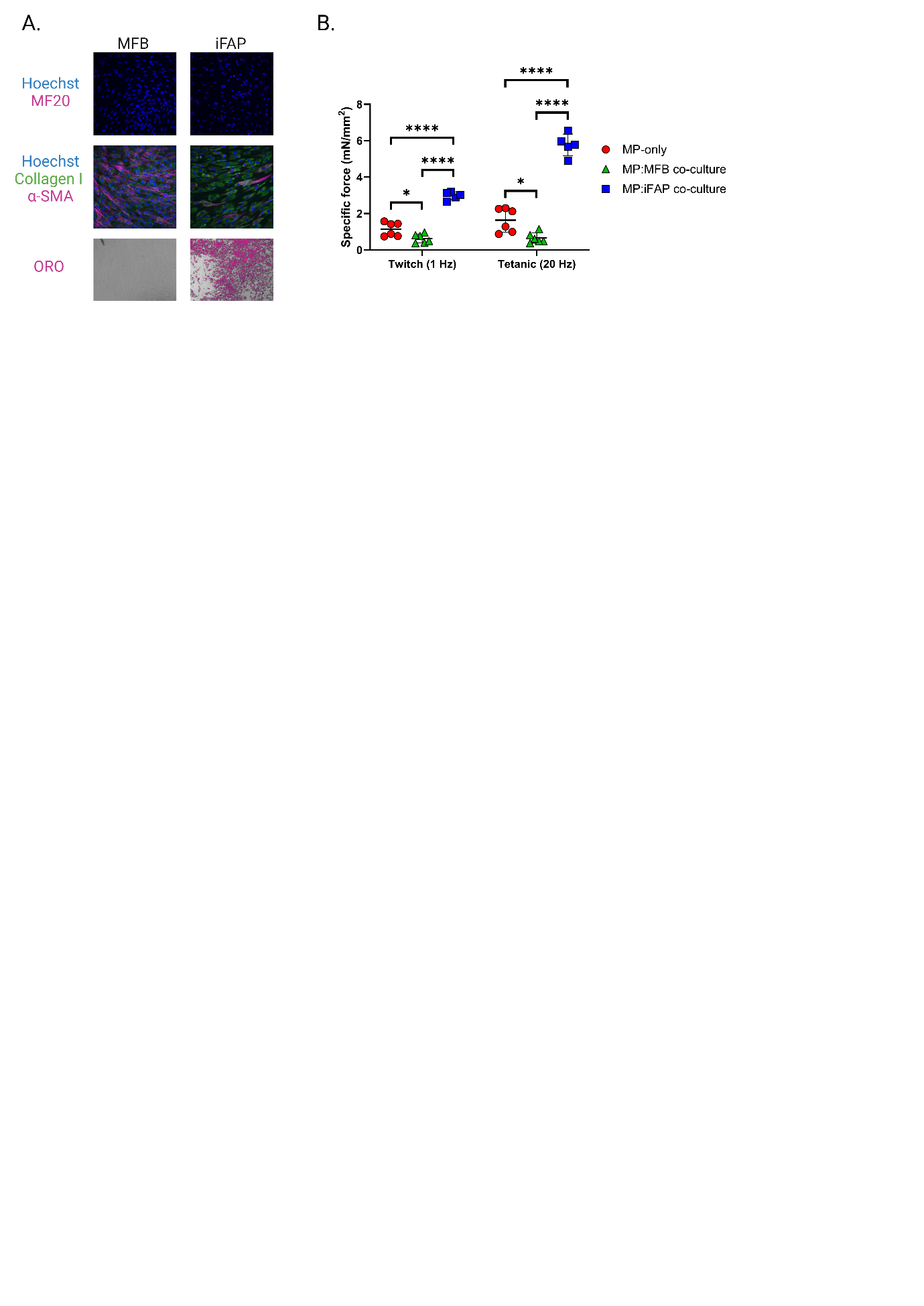


**Supplemental Figure 4. Improving contractile forces of 3D-TESMs is specific to iFAPs.** A) Both MFBs and iFAPs cannot differentiate into myotubes (MF20 staining). They both produce collagen 1 and a-SMA stress fibers (more visible in MFBs) and only iFAPs are able to differentiate into adipocytes (Oil red O staining). B) Specific force twitch and tetanic measurements, comparing 3D-TESMs consisting of 100% MPs versus 80% MPs + 20% MFBs versus 80% + 20% iFAPs. Analysis was performed at 7 days of differentiation, 3D-TESMs with MFBs showed the lowest contractile forces and 3D-TESMs with iFAPs were significantly stronger than the other conditions. For statistical analysis, a one-way ANOVA with Tukey correction for multiple comparisons was performed. Statistical significance was set at *P<0.05, ****P<0.0001. Error bars display the standard deviation. N=5-6, independent TESMs (biological replicates).


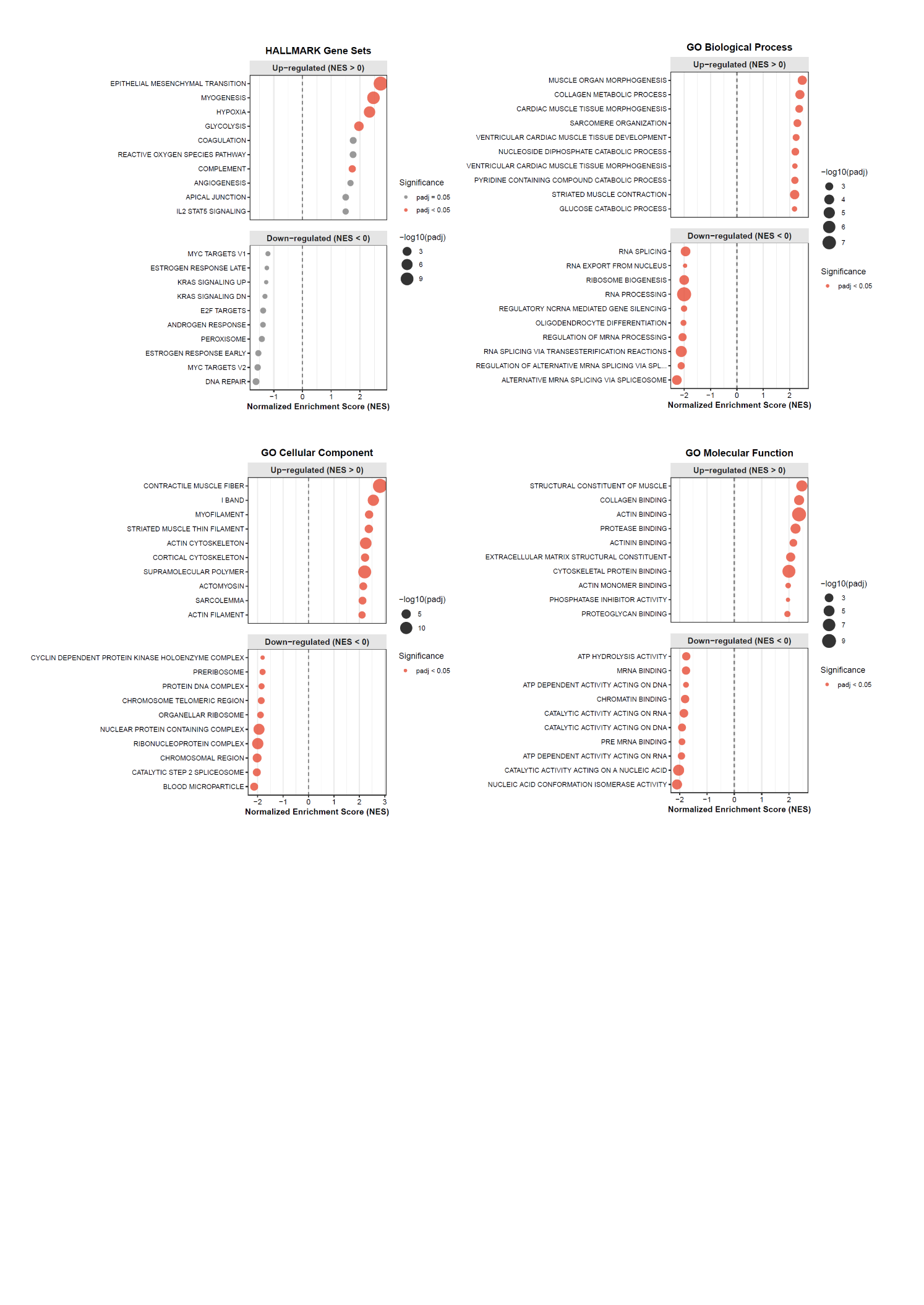


**Supplemental Figure 5. Gene Set Enrichment Analysis reveals pathway-level alterations in co-culture versus MP-only 3D-TESMs.** Gene Set Enrichment Analysis (GSEA) was performed using all 3,727 quantified proteins ranked by log2 fold change. Bubble plots display the top 10 positively enriched (highest NES) and top 10 negatively enriched (lowest NES) gene sets from four collections: MSigDB Hallmark gene sets (50 canonical pathways), Gene Ontology Biological Process, Gene Ontology, Cellular Component, and Gene Ontology Molecular Function. The x-axis shows Normalized Enrichment Score (NES), with positive values indicating upregulation and negative values indicating downregulation in co-culture 3D-TESMs. Bubble size represents -log10 (adjusted P-value). Red bubbles denote significantly enriched gene sets (adjusted P-value < 0.05); gray bubbles denote non-significant enrichment. Gene sets are ordered by NES within each panel.


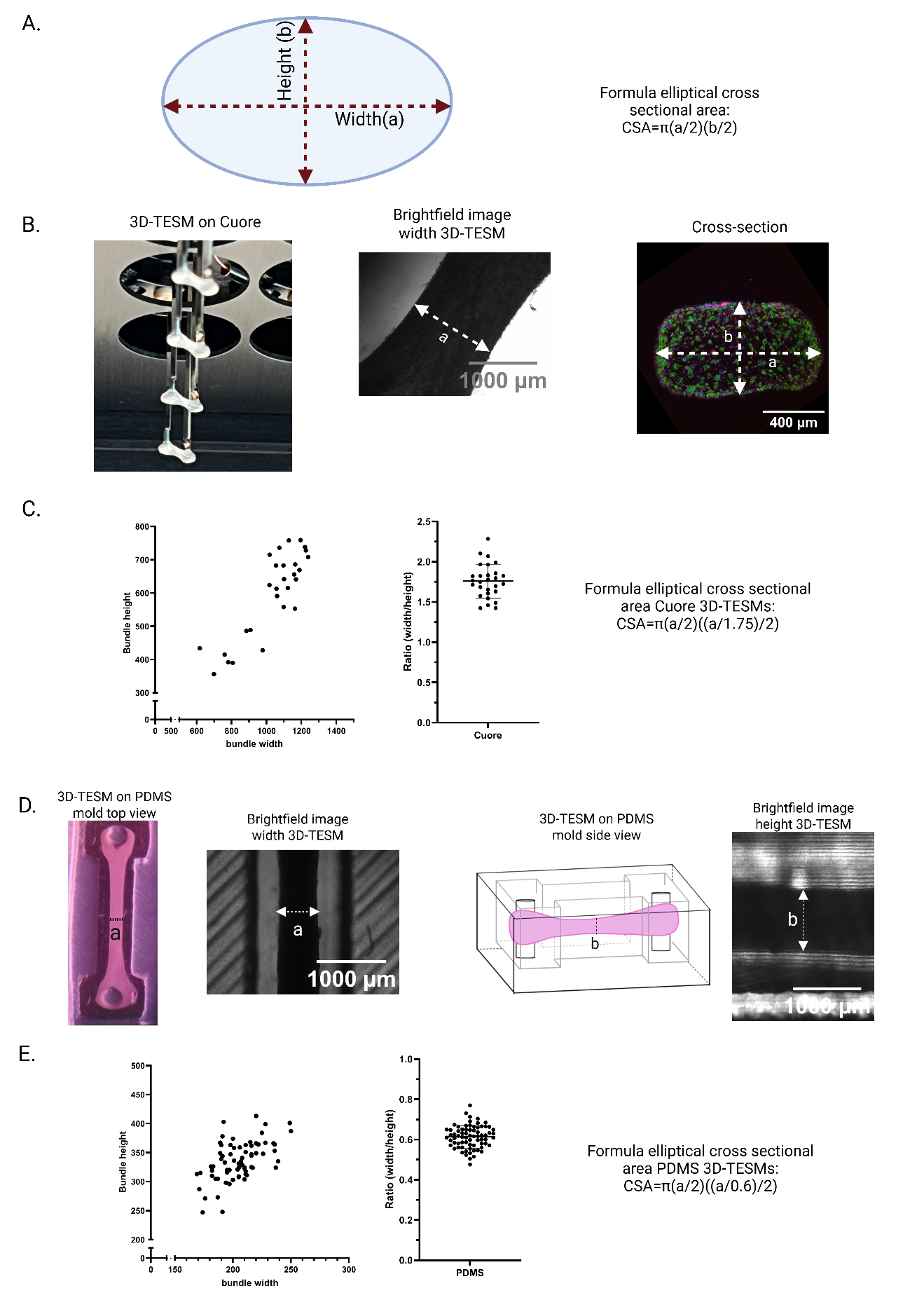


**Supplemental Figure 6. Cross sectional area (CSA) calculations for Cuore and PDMS platforms.** A) CSA estimated as elliptical shape from the width (a) and height (b) of the 3D-TESM. B) 3D-TESMs cultured on the Cuore platform showing the width and height measurements of a representative 3D-TESM. C) Width versus height distribution of 28 independent 3D-TESMs cultured on the Cuore platform. Measurements were performed on cross-sections. The resulting ratio results in a CSA formula based on only the width measurements specific to 3D-TESMs cultured on the Cuore platform. C,D) Width and height measurements of representative 3D-TESMs cultured on PDMS molds, resulting in a specific CSA formula.

**Supplemental Table 1. Protein abundance of samples used for proteome analysis.**

| SampleID | min_abundance | max_abundance | median_abundance |
| --- | --- | --- | --- |
| MP_only_3D_TESMs_1 | 8.1 | 380307 | 480.0 |
| MP_only_3D_TESMs_2 | 8.3 | 361915 | 463.2 |
| MP_only_3D_TESMs_3 | 5.9 | 338238 | 436.3 |
| Co_culture_3D_TESMs_1 | 9.4 | 441666 | 577.6 |
| Co_culture_3D_TESMs_2 | 11.4 | 476672 | 585.8 |
| Co_culture_3D_TESMs_3 | 8.5 | 485539 | 588.7 |
|  |  |  |  |
| # Accession | 3,727 |  |  |
| # Gene | 3,727 |  |  |
